# Investigating Ionic Diffusivity in Amorphous LiPON using Machine-Learned Interatomic Potentials

**DOI:** 10.1021/acsmaterialsau.4c00117

**Published:** 2025-02-05

**Authors:** Aqshat Seth, Rutvij Pankaj Kulkarni, Gopalakrishnan Sai Gautam

**Affiliations:** Department of Materials Engineering, Indian Institute of Science, Bengaluru 560012, India

**Keywords:** LiPON, solid-state electrolytes, machine learning, Li-ion batteries, interatomic potentials, interfaces

## Abstract

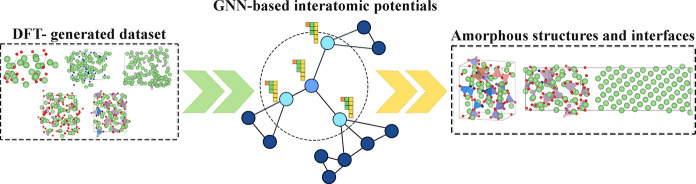

Due to its immense importance as an amorphous solid electrolyte in thin-film devices, lithium phosphorus oxynitride (LiPON) has garnered significant scientific attention. However, investigating Li^+^ transport within the LiPON framework, especially across a Li||LiPON interface, has proven challenging due to its amorphous nature and varying stoichiometry, necessitating large supercells and long time scales for computational models. Notably, machine-learned interatomic potentials (MLIPs) can combine the computational speed of classical force fields with the accuracy of density functional theory (DFT), making them the ideal tool for modeling such amorphous materials. Thus, in this work, we train and validate the neural equivariant interatomic potential (NequIP) framework on a comprehensive DFT-based data set consisting of 13,454 chemically relevant structures to describe LiPON. With optimized training (validation) energy and force mean absolute errors of 5.5 (6.1) meV/atom and 13.6 (13.2) meV/Å, respectively, we employ the trained potential to model Li transport in both bulk LiPON and across Li||LiPON interfaces. Amorphous LiPON structures generated by the optimized potential resemble those generated by *ab initio* molecular dynamics, with N being incorporated on nonbridging apical and bridging sites. Subsequent analysis of Li^+^ diffusivity in the bulk LiPON structures indicates broad agreement with prior computational and experimental literature. Further, we investigate the anisotropy in Li^+^ transport across the Li(110)||LiPON and Li(111)||LiPON interface, where we observe Li transport across the interface to be one order of magnitude slower than Li motion within the bulk Li and LiPON phases. Nevertheless, we note that this anisotropy of Li transport across the interface is minor, and we do not expect it to cause any significant impedance buildup. Finally, our work highlights the efficiency of MLIPs in enabling high-fidelity modeling of complex noncrystalline systems over large length and time scales.

## Introduction

The utilization of solid electrolytes in lithium (Li)-based energy storage technology is an active area of research as solid electrolytes allow for the use of lithium metal anodes, thereby improving the energy density and safety of next-generation Li-based batteries.^1−8^ Specifically, amorphous materials being used as solid electrolytes are particularly promising, given their wide compositional stability (or flexibility) that allows for a significant change in the ionic content without adverse phase transformations,^9^ their lack of grain boundaries that mitigates charge transfer impedance,^10^ and the absence of electrostatic or structural inhomogeneities^11^ that can result in the nucleation of dendrites.^12^ An example of an amorphous solid electrolyte is the lithium phosphorus oxynitride (of chemical formula Li_*x*_PO_*y*_N_*z*_ where *x* = 2*y* + 3*z* – 5), commonly referred to as LiPON, which has been demonstrated in thin-film energy storage devices.^13^ Synthesized first by Bates et al.,^14^ LiPON is typically made by incorporating N into Li_3_PO_4_ via radio frequency (RF) magnetron sputtering. The remarkable properties exhibited by LiPON are often attributed to the incorporation of N into the Li_3_PO_4_ structure, including electrochemical and mechanical stability,^15^ low electronic conductivity (10^–15^ to 10^–12^ S/cm),^16^ moderate ionic conductivity (3 × 10^–6^ S/cm),^17−19^ high critical current density (>10 mA/cm^2^),^20^ and excellent cyclability against lithium metal anodes.^21,22^ Despite the well-established properties of LiPON, the specific role of N in enhancing the performance of LiPON, especially on suppressing Li dendrite formation, still remains uncertain. The wide compositional range of LiPON, in addition to its amorphous structure, makes both experimental and computational investigations of the system challenging.

Prior analyses of the N 1s X-ray photoelectron spectroscopy (XPS) data on LiPON thin films have suggested that N atoms in LiPON cross-link by bonding to two or three phosphate tetrahedra, resulting in the formation of double-bridging (N_d_) and triple-bridging (N_t_) N sites, respectively.^23−28^ Such cross-linking by N leads to a “mixed anion effect”,^29^ which can provide Li^+^ with interconnected, low activation energy pathways, thus improving their diffusivity with respect to bulk, crystalline Li_3_PO_4_.^30^ Wang et al., based on X-ray diffraction and chromatography data along with the initial XPS data, proposed the presence of apical or nonbridging N atoms (N_a_), leading to isolated PO_3_N tetrahedra, besides a small amount of N_d_.^31^ Other studies have claimed that LiPON resembles metaphosphate glasses with extended chains of phosphate tetrahedra linked by N or O atoms or layered structures with Li and P rich regions,^32,33^ which does not match with the presence of isolated PO_4_ tetrahedra in the precursor phase of Li_3_PO_4_. Thus, there still exists substantial uncertainty regarding the local structure of the electrolyte.

Computational studies, employing tools such as density functional theory (DFT)-based calculations, ab initio molecular dynamics (AIMD), and classical molecular dynamics (MD) based on machine-learned interatomic potentials (MLIPs) have also been used to shed light on the structural features contributing to the enhanced electrochemical properties of LiPON. For example, Sicolo and Albe observed N atoms in the form of both N_d_ and N_t_ in their melt-quench generated amorphous LiPON structure, albeit with a stoichiometry (Li_1.25_PO_2_N_0.75_) more closely resembling bulk phosphate glasses than the thin-film solid electrolytes.^34^ Lacivita et al. observed N to be incorporated as N_a_ and N_d_ (and not N_t_) in their AIMD-generated LiPON structures^35^ and proposed that the N_d_ atoms densified the LiPON framework leading to the destabilization of Li^+^ and an improved Li mobility. Further, the authors found the N_a_:N_d_ ratio to increase as the Li content in LiPON increases, eventually resulting in all N occupying only N_a_ sites at a composition of Li_3.38_PO_3.62_N_0.38_. Subsequently, the authors proposed alternative assignments of existing experimental data that fully avoid assigning N to N_t_ sites, by using a combination of computational techniques and neuron scattering and infrared (IR) spectroscopy.^36^ Using solid-state nuclear magnetic spectroscopy (ssNMR) with AIMD, Marple et al. further explored the short-range environment of the phosphorus atoms in LiPON and reported four distinct phosphate tetrahedra configurations, namely, PO_4_^3–^, PO_3_N^4–^ (N_a_), P_2_O_6_N^5–^ (N_d_), and P_2_O_7_^4–^.^37^ Despite a multitude of studies on the amorphous nature of LiPON, Li transport in amorphous LiPON and across a Li||LiPON interface has not been probed in detail so far.

Given that amorphous systems often require large length and long time scales to sample the system dynamics well, classical MD powered by MLIPs is highly pertinent to model amorphous systems, given their high accuracy and low computational costs compared to DFT/AIMD.^38−41^ Typically, MLIPs are trained on a DFT-computed data set to mathematically approximate the potential energy surface (PES) of several configurations that a system can exhibit.^42^ Among the diverse set of MLIPs available, graph neural network (GNN)-based potentials, where the atomic structures are mapped onto graphs containing nodes (atoms) and edges (bonds) that are subsequently convoluted to incorporate short- and long-range interactions and invariant/equivariant symmetry constraints, have emerged as highly data-efficient architectures for deep learning PES of materials.^43−45^ The neural equivariant interatomic potential (NequIP)^46^ is particularly promising among GNN-based potentials since NequIP utilizes higher-order equivariant tensors that preserve translational, rotational, and permutational invariance,^45^ allowing it to build “flexible” potentials with high accuracy and fewer training structures compared to other MLIPs.

In this study, we develop a DFT and AIMD-computed LiPON-based data set and use it to train and optimize NequIP to model Li^+^ transport within the bulk amorphous framework and across Li||LiPON interfaces. We generate over 13,000 configurations for training and validating the NequIP model via DFT calculations on LiPON precursors, strained bulk structures, lattices with varying Li concentrations, slab/surface configurations, and AIMD simulations. Subsequently, we use the trained NequIP model to generate amorphous structures and probe Li^+^ diffusivity using MD simulations. Importantly, we find the predicted amorphous LiPON structures to resemble structures presented in prior AIMD-based simulations, highlighting NequIP’s accuracy in predicting structural features of LiPON. To investigate Li^+^ transport in LiPON-based thin-film devices, we simulate Li^+^ diffusion through bulk amorphous LiPON as well as across Li(110)||LiPON and Li(111)||LiPON interfaces. We observe Li^+^ diffusivity to be anisotropic across the Li||LiPON interface, varying by roughly 1 order of magnitude within the bulk and across the interface, with the degree of anisotropy dependent on the simulation temperature. We hope that our study and the potentials that we have constructed allow for further exploration of bulk LiPON and Li||LiPON interfaces and facilitate the utilization of MLIPs in the investigation of other amorphous solid electrolytes.

## Methods

### Data Set Generation

Our data set can be broadly divided into five categories of structures, which were generated using different procedures, namely, (i) strained, (ii) Li-rich, (iii) Li-poor, (iv) melt-quench, and (v) slab-based. Using an initial set of 19 different chemical systems (as listed in Table S1 of the Supporting Information), including elemental Li, Li_3_P, Li_3_N, LiPN_2_, Li_2_O_2_, and Li_3_PO_4_, we used the pymatgen^47^ package to generate various strained configurations, which can capture the local environment while Li^+^ diffuses within the amorphous LiPON framework. We induced hydrostatic (−10 to +9%), monoclinic (−8.65 to +8.65%), and orthorhombic (−10 to +10%) strains on the DFT-relaxed bulk structures, amounting to a total of 886 strained configurations. To capture the variations in the Li content, we generated 256 defective configurations, i.e., by replacing one O with a N in the unit cell as well as in a 3 × 2 × 2 supercell of Li_3_PO_4_. To balance the charge within the defective structures (i.e., one O replaced with a N), we enumerated symmetrically distinct ways to add a single lithium mimicking Li-rich conditions and also enumerated ways to remove both an O and a Li to model Li-poor concentrations. Additionally, we constructed 74 Li-rich structures by enumerating the removal of 1 P and the concomitant addition of 5 Li within Li_7_PN_4_ 2 × 2 × 2 supercells. To eventually model local environments that may be encountered in a Li||LiPON interface, we incorporated 1219 slab-based configurations from several chemistries, including elemental Li, Li_3_P, Li_2_O, and Li_3_N. The slabs were generated with a vacuum spacing of 20 Å and different slab thicknesses of 10–30 Å. Finally, to capture the amorphous nature of LiPON during training, we generated 11,000 amorphous structures using the melt and quench approach in AIMD simulations, resulting in a total data set of 13,454 configurations. We have made available the entire data set used for training and validating our NequIP model, as part of our GitHub repository (see the data availability section) with more details about the data set being available in the Supporting Information.

### Training and Validation

NequIP expresses the PES of a given structure through the summation of atomic energies, which, in turn, are functions of their corresponding local environment, while atomic forces are obtained as the gradient of the total potential energy. NequIP makes use of equivariant convolutions in 3D Euclidean space (or E(3)-equivariant)^48^ for modeling the interactions among its geometric tensor-based features. The convolution filter in NequIP is a product of equivariant and learnable radial functions and spherical harmonic functions.^49^ We split our overall 13,454 structure data set randomly in a 90:10 ratio for training and validation, respectively, where we optimized the hyperparameters to obtain the least mean absolute errors (MAEs) on atomic forces in the validation set. To investigate the effect of equivariance on the accuracy of the trained potential, we trained a NequIP model with the optimized hyperparameters but with the feature set restricted to only scalars. The final set of optimized hyperparameters is in Table S2 along with the final energy and force MAEs that we obtained on our train and validation sets. After constructing the optimized NequIP model, we performed melt-quench simulations using this model to generate amorphous LiPON structures and subsequently calculate Li^+^ diffusivity in bulk LiPON and across the Li||LiPON interface.

### DFT and AIMD Calculations

We used the Vienna ab initio simulation package (VASP)^50,51^ with the projector-augmented wave (PAW)^52,53^ potentials to generate the entire DFT-calculated data set. For treating the electronic exchange and correlation, we used the Perdew–Burke–Ernzerhof (PBE) functionalization of the generalized gradient approximation (GGA).^52^ We fixed the kinetic energy cutoff to 520 eV and sampled the Brillouin zone using Γ-centered Monkhorst–Pack *k*-point meshes of density 32/Å (i.e., a minimum of 32 *k*-points were sampled across a reciprocal space lattice vector of 1 Å^–1^) for relaxing all structures. For all structures, we relaxed the cell volume, cell shape, and ionic positions without symmetry constraints until the total energies and atomic forces converged within 10^–5^ eV and |0.03| eV/Å, respectively. We performed only a single self-consistent field (SCF) calculation for all strained configurations (until total energies converged within 10^–5^ eV). Additionally, we performed a single SCF calculation on the relaxed geometries of all non-AIMD structures at an energy cutoff of 400 eV, to ensure that our energy scales are comparable with the AIMD simulations (see below).

To generate the DFT-calculated amorphous training data set, we melted 2 × 2 × 2 supercells for the calculated ground-state structures for Li_3_PO_4_ and Li-rich defective structures by rapid heating until 3000 K, using AIMD simulations. Subsequently, we quenched the molten structures from 3000 K to approximately 0 K, at a rate of 250 K/ps. For Li_3_P, Li_2_O, and Li_3_N, we followed a similar melt-quench approach where we heated the corresponding relaxed supercells consisting of 144, 24, and 108 atoms up to 2000, 2000, and 1000 K, respectively. Our choice of temperatures up to which we heated the systems considered was based on the corresponding melting points of the compounds, namely, 1110 K for Li_3_PO_4_, 742 K for Li_3_P, 1711 K for Li_2_O, and 1087 K for Li_3_N. We noticed significant amorphization of Li_3_N even though we did not heat it to temperatures beyond its melting point (Figure S1). For all AIMD simulations, we used the NVT ensemble with a Nose–Hoover thermostat,^54−56^ a time step of 2 fs, and a kinetic energy cutoff of 400 eV. We used a lower kinetic energy cutoff in our AIMD calculations to reduce computational costs.

### Generating Amorphous LiPON

We used the trained NequIP potential to generate the “equilibrated” and amorphous LiPON structures using the large-scale atomic/molecular massively parallel simulator (LAMMPS)^57^ package. The initial LiPON configuration for the LAMMPS simulations was generated by taking a 2 × 2 × 2 Li_3_PO_4_ supercell of 128 atoms (i.e., 16 formula units), replacing five random oxygen atoms with nitrogen atoms and balancing the charges by removing Li and O atoms, leading to a final composition of Li_2.94_PO_3.5_N_0.31_. To create the equilibrated structures at different temperatures (i.e., structures not necessarily intended to become amorphous), we subjected the initial crystalline LiPON structure to NVT simulations of 100 ps, with a time step of 2 fs at temperatures of 600, 900, 1200, and 1500 K. For generating amorphous LiPON, we started by melting the initial LiPON structure under NVT at 2000 K for 10 ps, with a time step of 1 fs, followed by a quench from 2000 to 250 K at a rate of 250 K/ps.

### Construction of the Li||LiPON Interface

The choice of Li(110) and Li(111) is motivated by their high stability (as indicated by the low calculated surface energies of 0.0309 and 0.0330 eV/Å^2^, respectively) and low lattice parameter mismatch between them and our amorphous LiPON structure. We chose the 2 × 2 × 1 supercell of the Li(110) slab to interface with the MLIP-based melt-quench LiPON structure. Subsequently, we modified the LiPON structure such that its lattice parameters match those of the 2 × 2 × 1 Li(110) slab along the *a* and *b* directions. This transformation of the LiPON structure leads to a contraction of 1.74% of the surface area along the *a–b* plane. To offset this contraction and preserve the initial volume of the LiPON structure, we expanded the LiPON structure along the *c* direction by 1.39%. The Li(110) slab was given a thickness of 19.5 Å to distinguish the interfacial behavior from that of the bulk, leading to a Li(110) slab consisting of 81 atoms. The total interface structural model thickness was fixed at 36.5 Å, with a gap of 2 Å between the Li(110) slab and the LiPON slab. Additionally, we created a larger Li(110)||LiPON interface, consisting of ∼1300 atoms, to demonstrate that NequIP-based simulations can be performed in larger systems as well (see the schematic in Figure S8). A similar matching approach was used to generate the Li(111)||LiPON interface with a 2 × 2 × 1 supercell of the Li(111) slab interfaced with the MLIP-based melt-quench LiPON structure.

### Li^+^ Diffusivity

We modeled Li^+^ diffusivity within the NequIP-generated amorphous LiPON structures via NVT ensemble simulations of a minimum of 100 ps with a time step of 5 fs at different temperatures starting from 300 to 900 K at intervals of 100 K each, using LAMMPS powered by our NequIP model. Similarly, we modeled Li^+^ motion across the different Li||LiPON interfaces for 75 ps with a time step of 5 fs at different temperatures. The amorphous LiPON structures consist of 124 atoms while our Li(110)||LiPON and Li(111)||LiPON structures consist of 205 and 196 atoms each. We also calculate diffusivity in a much larger Li(110)||LiPON interface consisting of 1316 atoms, generated by matching a 2 × 2 × 2 supercell of our amorphous LiPON structure with a 4 × 4 × 1 supercell of the Li(110) slab in a similar manner as discussed above. In both amorphous and interfacial systems, ionic diffusivity (*D*) was calculated as the slope of the mean square displacement (MSD) of Li atoms over the time interval (Δ*t*), as given by eq 1, where *d* refers to the dimensionality of the system. We measure the MSD by averaging over the MSD of each Li ion within the entire simulation, i.e., our calculated *D* corresponds to the tracer diffusivity of Li ions.
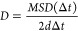
1

However, a simple slope of the MSD versus Δ*t* does not account for the ballistic and vibrational motion of the ions. Hence, we followed the procedure proposed by He et al.,^58^ which neglects the time step increments until the MSD reaches a value of 0.5*a*^2^, where *a* denotes the average distance between two neighboring Li sites (≈3 Å). Also, we ensured the linearity of MSD versus Δ*t* while estimating *D*. Using the calculated diffusivity, we further calculated the ionic conductivity in amorphous LiPON via the Nernst–Einstein relation, as in eq 2.
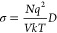
2where *V*, *N*, *q*, *k*, and *T* denote the volume of the system, the number of mobile Li ions, the charge on a Li^+^, the Boltzmann constant, and the temperature, respectively.

## Results

### AIMD Simulations

**Figure 1:**
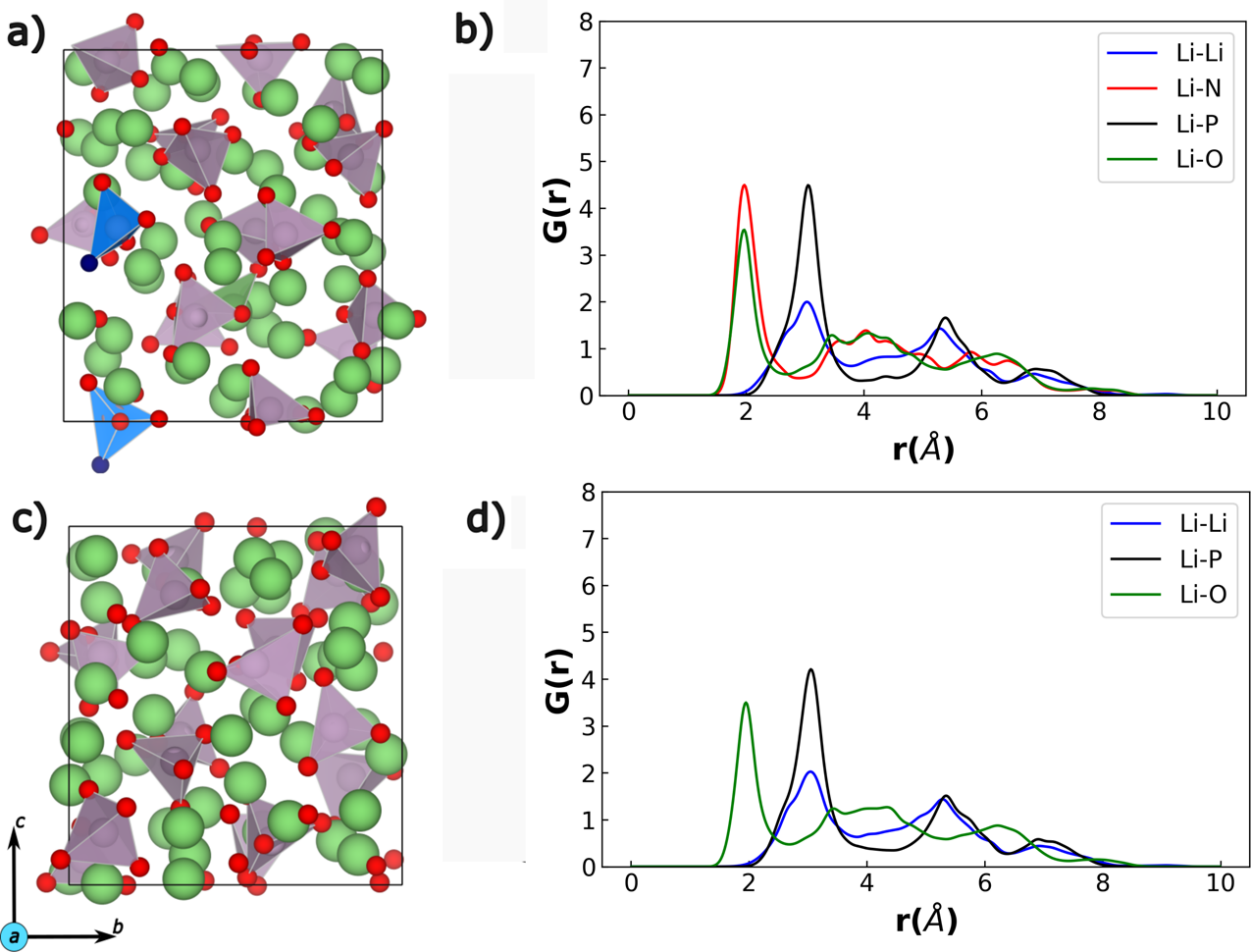
Sample AIMD-generated amorphous Li-rich LiPON structure (panel a) and amorphous Li_3_PO_4_ structure (panel c). Green, red, blue, and violet spheres are Li, O, N, and P, respectively. The PO_4_ tetrahedra are in violet color with blue tetrahedra indicating the presence of N occupying the N_a_ sites in P-based groups. The RDFs of LiPON and Li_3_PO_4_ are plotted in panels b and d, respectively.

Examples of the AIMD-generated amorphous Li-rich LiPON and Li_3_PO_4_ structures along with their respective time-averaged radial distribution function (RDF) plots are shown in Figure 1. Note that the structures are taken at 250 K with the RDFs being time-averaged as the system equilibrates at 250 K after being fully quenched. The green and red spheres in panels a and c of Figure 1 indicate Li and O atoms, while the purple polyhedra indicate PO_4_ groups. N atoms in the LiPON structure are highlighted by blue spheres and the PO_3_N tetrahedral groups, which contain N, are colored in blue in Figure 1a. In panels b and d, blue, black, green, and red lines indicate Li–Li, Li–P, Li–O, and Li–N neighbors (or bonds), respectively. Sample RDFs from melt-quench AIMD simulations of Li_3_N, Li_3_P, and Li_2_O are compiled in Figure S1, with Li_3_N and Li_3_P showing distinct signatures of amorphous phases.

Importantly, the lack of uniform and periodic sharp peaks in the RDF plots (panels b and d of Figure 1) indicates that the structures are completely disordered after the melt-quench process within our AIMD simulations. Further, the broad peaks of all types of Li-based neighbors beyond 4 Å in both LiPON and Li_3_PO_4_ indicate the lack of any long-range order, a typical signature observed in amorphous structures. Comparing the two calculated RDFs, we notice that the distribution of bonds remains largely unaffected even after the incorporation of N in amorphous Li_3_PO_4_, with Li–Li bonds showing minor variations. Notably, we observe the distribution of the Li–N bonds in LiPON to closely follow that of Li–O bonds (in LiPON/Li_3_PO_4_), especially between 1 and 4 Å, indicating the occupancy of N primarily among the N_a_ sites instead of N_d_ and N_t_ sites (see Figure 1a), which results in similar Li–N distances compared to Li–O. The sharper Li–N peaks in LiPON compared to the Li–O peaks indicate the presence of excess Li atoms near the N center, to compensate for the larger negative charge of the N^3–^ compared to O^2–^. Thus, our melt-quench AIMD simulations have successfully created snapshots of amorphous local environments that can be present in the actual LiPON phase, which should result in an accurate NequIP model.

### Optimized Potential

Using the set of optimized hyperparameters in Table S2, we trained two different NequIP models, one using an equivariant tensor to generate the feature set during training (referred to as “l_max = 2” within the NequIP architecture) and the other using invariant scalars (l_max = 0). Expectedly, our potential trained with l_max = 2 features displayed significantly lower training energy and force MAEs, of 5.5 meV/atom and 13.6 meV/Å, respectively, compared to the model with l_max = 0 features (energy and force MAEs of 22.3 meV/atom and 101.9 meV/Å, respectively). The lower training errors on the l_max = 2 model are attributed to the equivariant tensor features better capturing the local environments sampled by the model than the invariant scalars. Further, both the l_max = 2 and l_max = 0 models displayed consistently lower force errors on the validation set compared to the training set, with specific MAEs of 6.1 (16.1) meV/atom and 13.2 (95.4) meV/Å across energies and forces, respectively, for the l_max = 2 (l_max = 0) model. The lower validation errors suggest that our models are likely not overfit on the training data. Given that the l_max = 2 model is more accurate than l_max = 0, we used l_max = 2 for further analysis throughout the rest of the manuscript.

**Figure 2:**
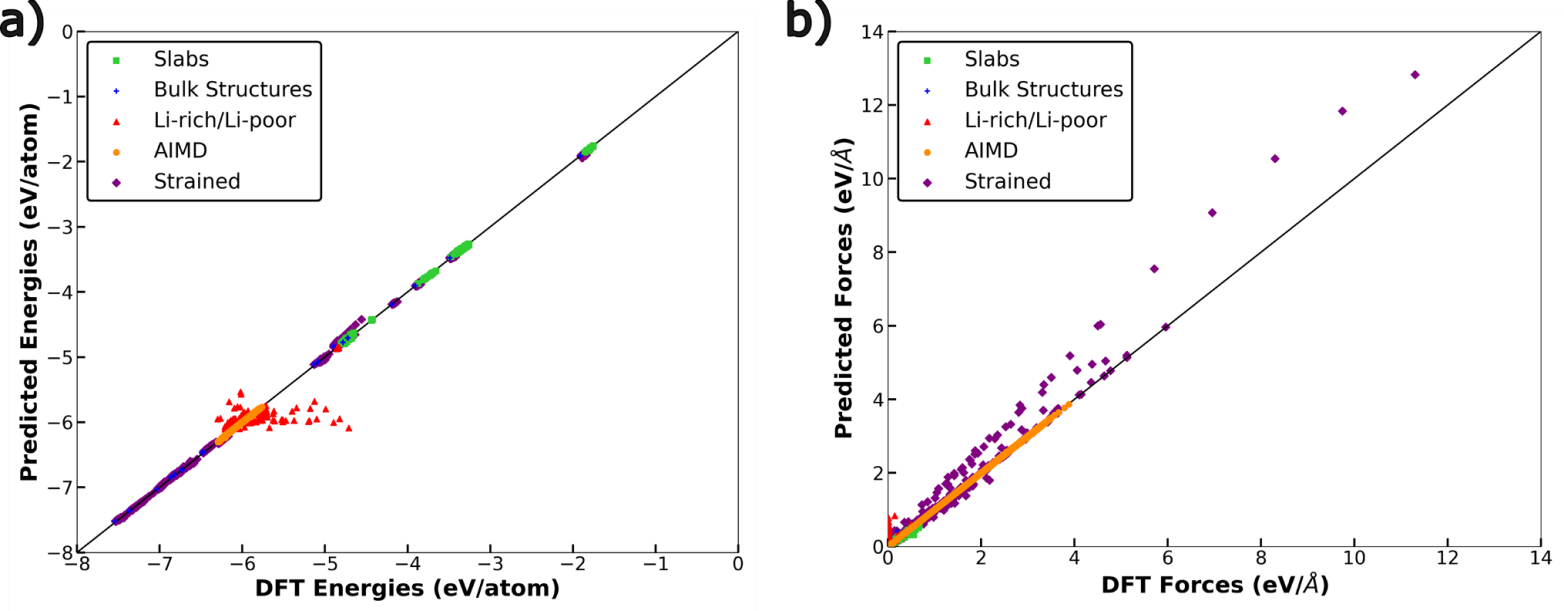
Parity between NequIP-predicted and DFT-calculated per atom energies (panel a) and atomic forces across the entire data set (panel b). The different symbols indicate the different subsets of the data set.

Figure 2 displays the parity between the NequIP (optimized l_max = 2 model) predicted energies and DFT-calculated energies (panel a) and atomic forces (panel b) for the complete data set (i.e., training and validation). The symbols in both plots indicate different subsets of the training set, namely, green squares for slabs, blue diamonds for the bulk stoichiometric phases, red triangles for the defective structures, orange circles for the AIMD simulations, and purple diamonds for the strained configurations. Overall, the NequIP-predicted energies are in strong agreement with DFT calculations, with the exception of the defective (i.e., Li-rich/Li-poor) data set. Similarly, the NequIP-predicted atomic forces are in good agreement with the DFT-calculated values, with the exceptions of the defective and strained data sets. The lack of agreement in predicted versus calculated forces in the strained subset is likely due to the large forces that are generated with the application of strain with our NequIP model underestimating the calculated values. While the reason for disagreement between NequIP-predicted and DFT-calculated energies and forces within the defective data set is unclear, we hypothesize that small perturbations in local bonding environments within these defective structures are resulting in larger variations in energies and forces, a phenomenon not fully captured by the NequIP model. Nevertheless, the overall training and validation errors exhibited by our NequIP model are close to those observed in the literature,^35^ across a wider and more diverse training set, suggesting that our model is robust enough in modeling the amorphous LiPON energy landscape.

### Amorphous LiPON

**Figure 3:**
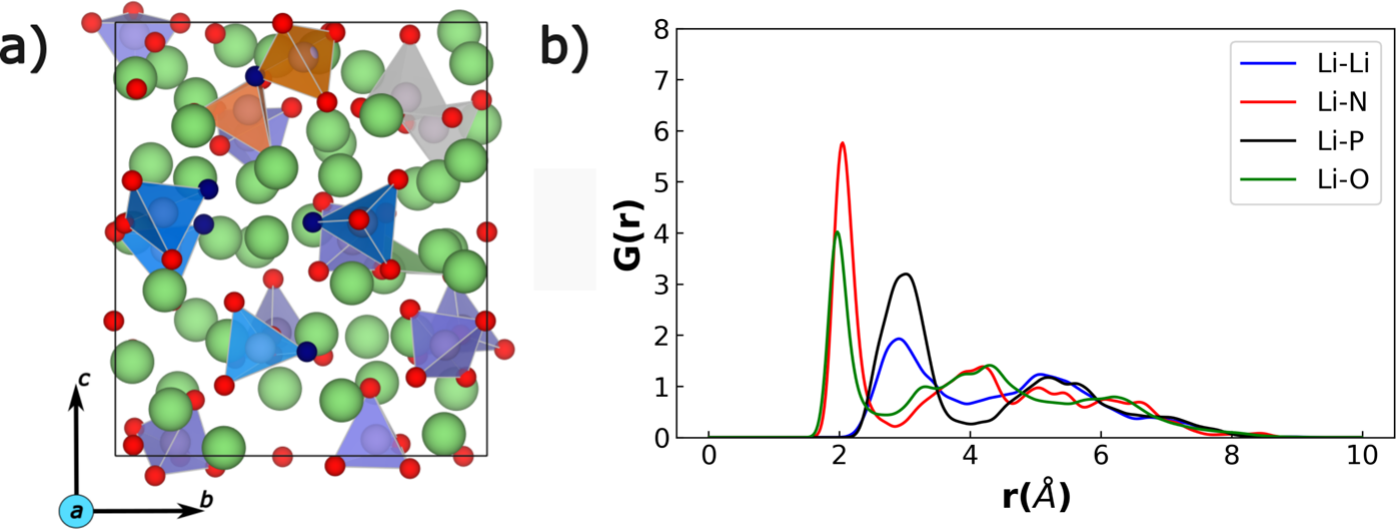
(a) Example NequIP-generated amorphous LiPON structure at 250 K generated with a composition of Li_2.94_PO_3.5_N_0.31_ via melt-quench MD simulations. Notations used are similar to those in Figure 1. Different P-based groups are highlighted with different colors: violet tetrahedra are PO_4_^3–^, blue tetrahedra are PO_3_N^4–^, gray polyhedra denote P_2_O_7_^4–^, and orange polyhedra are P_2_O_6_N^5–^ groups. (b) Corresponding time-averaged RDF of the generated LiPON structure.

Using the optimized NequIP (l_max = 2) potential, we generate amorphous LiPON structures using the melt-quench MD approach. An example of a generated amorphous LiPON configuration (consisting of 124 atoms and a composition of Li_2.94_PO_3.5_N_0.31_) that was melted at 2000 K and quenched to 250 K is displayed in Figure 3a, along with its RDF in Figure 3b. The notations used in Figure 3 are similar to those used in Figure 1, with the violet, blue, dark green, and orange tetrahedra indicating the PO_4_^3–^, PO_3_N^4–^, P_2_O_7_^4–^, and P_2_O_6_N^5–^ groups, respectively. RDFs of structures melted to 600, 900, 1200, and 1500 K and quenched to 250 K are displayed in Figure S2, while RDFs of structures equilibrated at 600, 900, 1200, and 1500 K and subsequently quenched to 250 K are displayed in Figure S3.

From the structural snapshot in Figure 3a, we observe the nitrogen atoms to be incorporated within the LiPON framework mostly as N_a_ (blue polyhedra), with few of the N sitting on N_d_ sites bridging two phosphate groups together (orange polyhedra). This matches prior experimental analysis by Wang et al.^31^ as well as the AIMD-based studies of Lacivita et al.^35,36^ Moreover, we also observe phosphate tetrahedra linked by oxygen, resulting in the formation of P_2_O_7_^4–^ groups (gray polyhedra), akin to the observations made by Marple et al.^37^ Importantly, we do not observe any triply coordinated N sitting on N_t_ sites in any of our melt-quench simulations, suggesting that it is unlikely to find N adopting this local coordination environment, which is in contrast to the initial experimental studies of Bates et al.^18^ Nevertheless, the lack of N on N_t_ sites is consistent with subsequent experimental and computational studies,^31,36,37^ highlighting that our potential is generating reliable amorphous structures.

In terms of the RDFs (Figure 3b), we observe broad peaks beyond 4 Å for all types of neighbors to Li atoms, indicating the lack of long-range order, similar to our observation in AIMD simulations as well (Figure 1). Different from our AIMD simulations, we do observe sharper Li–O and Li–N peaks at ∼2 Å, suggesting the formation of strong local order (or bonding) of Li atoms with nearby anions. Also, the Li–P RDF displays a broad shoulder and a peak toward ∼2.9 Å, suggesting the existence of short-range order between the Li atoms and P-based groups. The differences between our AIMD and NequIP-based MD simulations can be primarily traced to the extent of equilibration done at the quenched and differences in nitrogen concentration, with the MD simulations allowing the formation of local anionic clusters surrounding the Li atoms. With respect to quenching to higher temperatures (e.g., quenching to 600 K instead of 250 K), we observe a larger degree of short-range disorder when quenching to higher temperatures compared to 250 K, with the Li–O, Li–N, and Li–P peaks being significantly wider in these cases.

### Li Diffusivity in Amorphous LiPON

**Figure 4:**
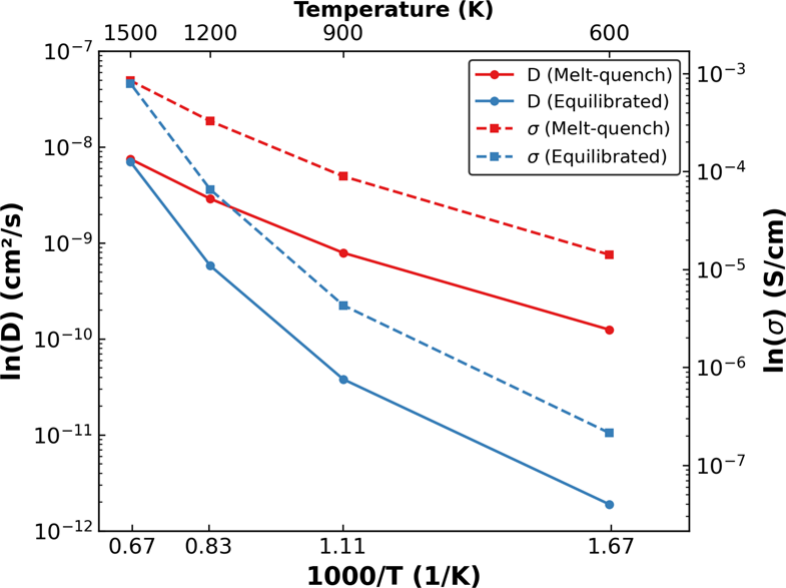
Li^+^ diffusivity and ionic conductivity measured against temperature, resulting from MD simulations conducted using the optimized potential.

We calculate the Li^+^ diffusivity (and associated conductivity) in both the melt-quench and equilibrated LiPON configurations generated by the optimized NequIP model at 600, 900, 1200, and 1500 K, as the elevated temperatures allow a decrease in the simulation time required to capture Li migration events. While the resultant natural logarithmic values of *D* (in cm^2^/s) and σ (S/cm) values are plotted in Figure 4 as a function of (inverse of) temperature, solid (dashed) red and blue lines in Figure 4 indicate the *D* (σ) values calculated in melt-quench and equilibrated structures, respectively. Given that *D* and σ are proportional to each other (see eqs 1 and 2), we observe similar trends in our calculated *D* and σ.

Our calculated *D* (and σ) values are consistently higher in the melt-quench structures (red lines in Figure 4) compared to the equilibrated structures (blue lines), except at the highest temperature simulated (i.e., 1500 K). For example, *D* in melt-quench LiPON is ∼2 orders of magnitude higher (1.25 × 10^–10^ cm^2^/s versus 1.89 × 10^–12^ cm^2^/s) than equilibrated LiPON. We can attribute the amorphous nature and the lack of significant long-range ordering (see Figures S2 and S3) to be a contributing factor to the higher *D* (and σ) observed in the melt-quenched structures compared to equilibrated structures, which are in line with experimental observations of superior Li conductivity in amorphous LiPON versus crystalline Li_3_PO_4_.^27,59^ In these structures, the amorphization leads to an orientational disorder in the phosphate tetrahedra destabilizing the Li ions.^19^ The convergence of our calculated *D* (and σ) values at 1500 K for both the melt-quench (7.5 × 10^–9^ cm^2^/s) and equilibrated (7.0 × 10^–9^ cm^2^/s) structures highlights that both structures become equally disordered at higher temperatures and exhibit similar local environments (see Figures S2 and S3), thus resulting in similar *D* (and σ). The higher Li diffusivity in LiPON compared to amorphous Li_3_PO_4_^60^ can be attributed to the presence of N that facilitates Li migration. Finally, we also calculate the room-temperature *D* for the melt-quench LiPON configuration and observe our reported *D* to be qualitatively similar to the *D* values reported by Lacivita et al.^35^ (1.08 × 10^–11^ cm^2^/s vs 7.00 × 10^–10^ cm^2^/s) at room temperature while our calculated σ matches the experimental σ at 298 K^27^ (1.22 × 10^–6^ S/cm vs 3.3 × 10^–6^ S/cm), suggesting that our NequIP model is able to provide qualitatively accurate trends with reasonable quantitative accuracy.

### Interface Model and Li Diffusivity

**Figure 5:**
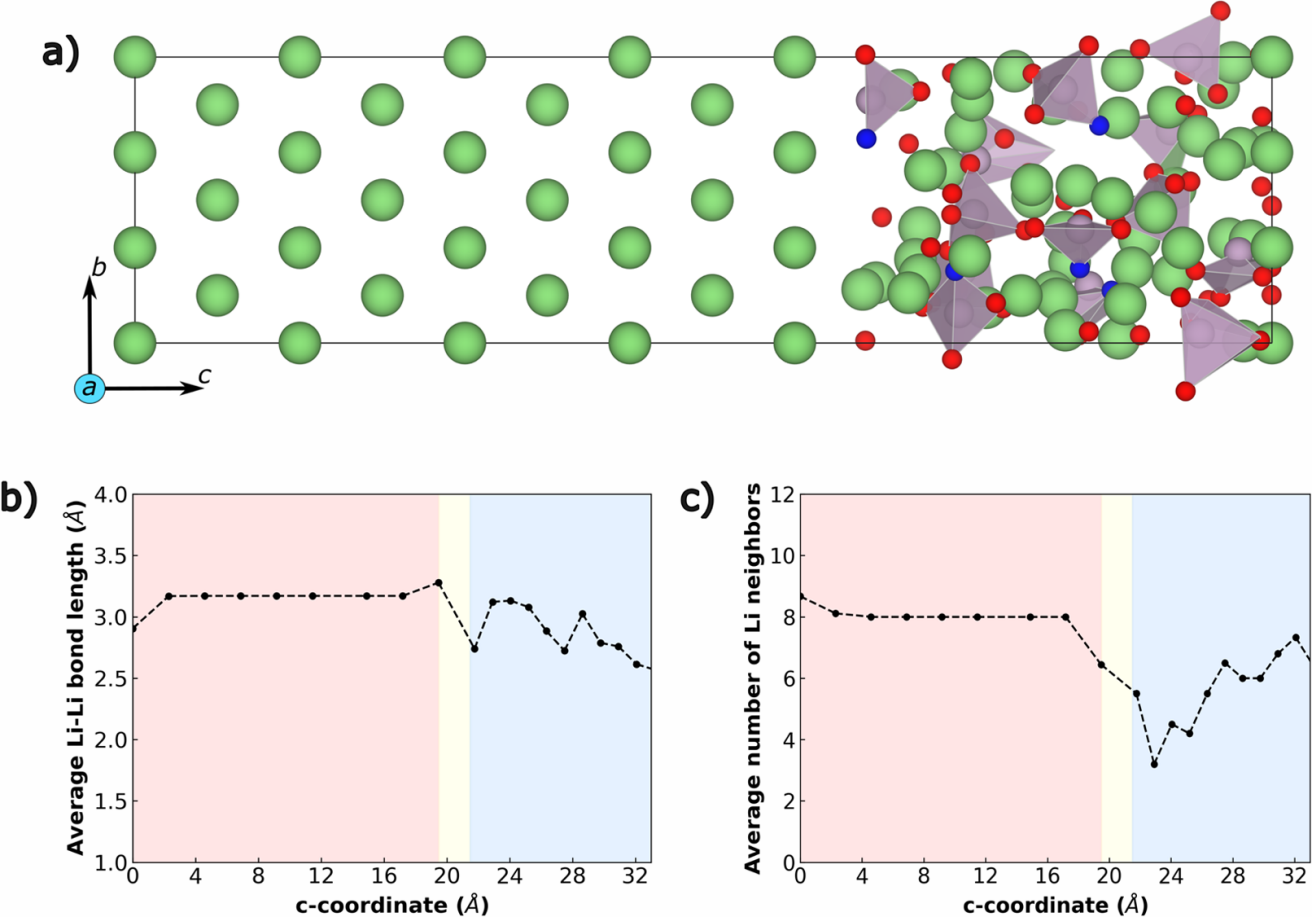
(a) Li-(110)||LiPON interface. (b) Variation in the average Li–Li bond length and (c) variation in the average number of Li neighbors along the *c*-axis.

Figure 5a shows the generated Li(110)||LiPON interface (for the Li(111)||LiPON interface, see Figure S5), with bulk Li metal constituting the “left” portion of the structure and bulk LiPON (generated via melt-quench) constituting the “right” portion. Green, purple, red, and blue spheres are Li, P, O, and N, respectively. Panels b and c of Figure 5 plot the average Li–Li bond length and the average number of Li neighbors to a given Li atom across the structure. Note that we average the bond length and number of Li neighbors at a given *c*-axis value (i.e., averaged across an *a–b* plane), where the *c*-axis is perpendicular to the interface. The pink, blue, and yellow shades in Figure 5b,c indicate regions of bulk Li, bulk LiPON, and the interface (i.e., the transition from Li to LiPON), respectively.

Importantly, we observe the average Li–Li bond distances and average number of Li neighbors to be uniform within the bulk Li region (shaded pink regions), highlighting the crystalline nature of body-centered cubic Li metal. The decrease (increase) in bond distances (number of neighbors) in the bulk Li region close to zero value of the *c*-axis is due to periodic boundary conditions utilized in our calculations. Across the transition region (shaded yellow regions), we observe sharp changes in both the Li distances and neighbors. Further, both bond distances and Li neighbors exhibit nonmonotonic trends within bulk LiPON (blue shaded regions), attributable to the amorphous nature of the structure.

**Figure 6:**
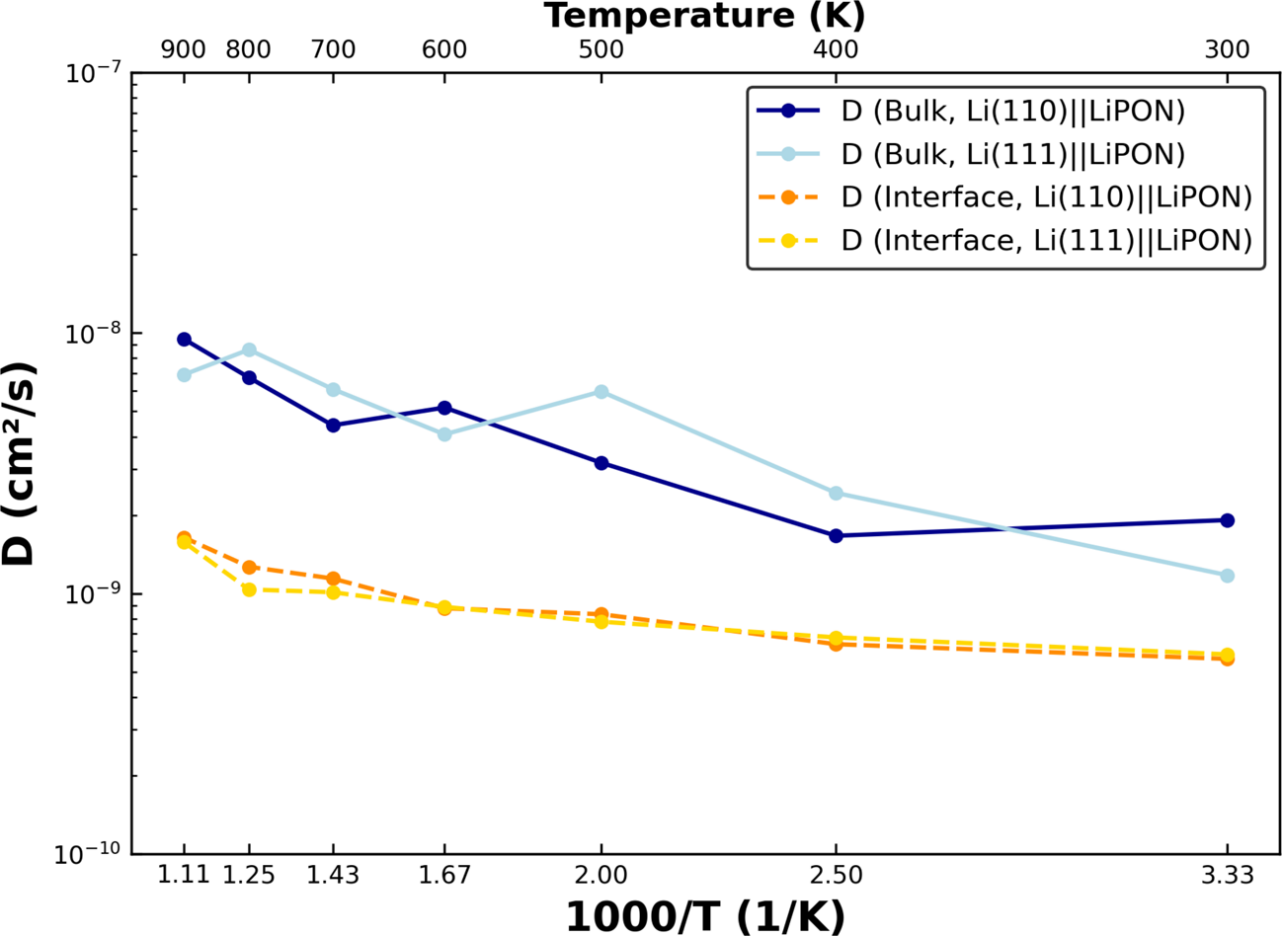
Variation of Li^+^ diffusivity through bulk phases and across the interfacial (transition) region of the Li(110)||LiPON and Li(111)||LiPON interface calculated at 300, 400, 500, 600, 700, 800, and 900 K.

The NequIP-calculated *D* of the Li(110)||LiPON and the Li(111)||LiPON interface is plotted in Figure 6, where we distinguish between *D* in the bulk phases (i.e., bulk Li and bulk LiPON, red lines) and across the transition region (or interface, blue lines) between Li and LiPON. Specifically, we calculate the *D* along the *c*-direction (i.e., perpendicular to the interface) and across the transition region to quantify Li transport from bulk Li to bulk LiPON (and vice versa), which in turn should correspond to how conductive to Li is the Li(110)||LiPON interface. Thus, the calculated *D* in the bulk phases correspond to Li migrations along *a*–*b* planes (i.e., parallel to the interface). The variation of the MSD of Li with Δ*t* at 300, 600, and 900 K are compiled in Figure S4.

Importantly, we find *D* in the bulk phases to be higher than the transition region (∼10^–8^ cm^2^/s versus 10^–9^ cm^2^/s), which is expected given that Li is known to diffuse reasonably well in its bulk metallic state and within amorphous LiPON. This difference in *D* between the bulk and the interface can be attributed to the different mechanisms in the two cases: vacancy-based hopping in the bulk phases while interfacial hopping need not be strictly based on vacancies. Thus, the vacancy-based hopping mechanism within the bulk can allow for greater diffusivity compared with transport across an interface. Across the transition region, the drop in *D* that we observe is marginal (i.e., 1 order of magnitude) and should not significantly affect the transport of Li from one bulk phase to another. However, our calculated *D* are lower than reported values across the Li(100)||Li_3_P interface (∼10^–6^ to 10^–7^ cm^2^/s) and are similar in magnitude to Li(110)||Li_2_S and Li(110)||LiCl interfaces (∼10^–7^ to 10^–9^ cm^2^/s).^61^ Given that Li interfaces with Li_2_S and LiCl are known to be poor for Li transport, resulting in an impedance increase in argyrodite-type electrolytes,^61^ we expect the Li||LiPON interfaces to also be active only under low-rate conditions, in agreement with the usage of LiPON in thin-film devices.^62^ Thus, further changes in composition or preconditioning of the Li||LiPON interface may be deployed for improving the power performance of LiPON electrolytes in practical devices. We find both the interfacial (2.85 × 10^–9^ cm^2^/s vs 5.608 × 10^–10^ cm^2^/s) as well as the bulk *D* values (4.07 × 10^–9^ cm^2^/s vs 1.193 × 10^–9^ cm^2^/s) for the larger Li(110)||LiPON supercell to be marginally higher than the smaller Li(110)||LiPON interface at 300 K, indicating our potential’s ability to be used for large-scale interfacial studies.

## Discussion

LiPON’s excellent electrochemical properties when compared to crystalline Li_3_PO_4_, including its ability to suppress cycle life degradation and dendrite formation over thousands of charge–discharge cycles, have made it a highly appealing solid electrolyte in thin-film devices. In this study, we have created a DFT-calculated data set of over 13,000 configurations, which spans the different local environments that may be encountered in an amorphous LiPON structure. We used the DFT data set to train an equivariant NequIP potential and in turn used the NequIP model to describe Li transport in bulk amorphous LiPON and across a Li(110)||LiPON interface. Specifically, we generated bulk amorphous LiPON structures of composition Li_2.94_PO_3.5_N_0.31_ using melt-quench MD simulations and via an equilibration approach that retained a higher degree of long-range order. Importantly, we found N to occupy the N_a_ and N_d_ sites only and did not observe any occupation of N_t_ sites. Further, our calculated Li diffusivities (and conductivities) in bulk LiPON are in qualitative agreement with previous studies, while we observe Li diffusivity across a Li(110)||LiPON interface to be ∼1 order of magnitude slower than the bulk phases. We hope that our work highlights the high accuracy, transferability, and efficiency of equivariant GNN-based MLIPs and motivates the utilization of these architectures in studying other complex amorphous structures.

The choice of data set plays a crucial role in the training and validation of any MLIP model. It is important for the data set to contain a diverse set of chemically relevant configurations and local bonding environments, enabling the MLIP model to learn the PES effectively. In this regard, we observe NequIP to be highly data-efficient owing to its equivariant GNN-based architecture, which allows for features to be propagated beyond the chosen cutoff radius via message passing. With a data set of just over 13,000 DFT and AIMD-generated configurations, the NequIP models not only achieve training energy and force errors (MAE) similar to what is typical of DFT but also show remarkable resistance to overfitting. Additionally, our data set could be used to train other MLIPs such as CHGNet,^63^ Allegro,^64^ DimeNET,^65^ and MACE^66^ to benchmark the performance of different GNN-based architectures. In any case, there do exist certain gaps in our training data set, which when supplemented with additional data could lead to even better potentials. For example, prior studies have indicated the Li-LiPON interface to be passivized by the presence of decomposition products such as Li_2_O, Li_3_N, Li_3_P, and Li_3_PO_4_.^67^ While AIMD-based configurations and individual slabs of these systems are part of our training data set, their interfaces with pure Li or LiPON, which are computationally expensive to compute, are lacking. Thus, expansion of the data set used in this work will result in better insights and more accurate predictions of the kinetics of the Li-LiPON system.

Along with the data set limitations, improvements in hyperparameter tuning could further improve the accuracy and usage of the trained potentials. One bottleneck in hyperparameter tuning and the general process of training MLIPs, is the computational cost of training. For instance, though NequIP’s potentials are highly data-efficient due to their use of equivariant tensors, their utilization of equivariant tensor-based features leads to an exponential increase in the required training time. Training on one NVIDIA Tesla V100 16 GB GPU card (with 192 GB of RAM and no hyperthreading), we observed training sessions to take as long as 36 h with tensor features, while using only invariant scalar features typically takes only a few minutes of training. However, we have shown that NequIPs with scalar features have significantly higher training and validation errors compared with NequIPs with tensor features. Hence, there exists a need for computationally optimizing the MLIP architectures, such that the models can be trained quickly.

Experimentally, Li^+^ diffusivity is significantly affected by the percentage of crystallinity, experimental conditions, and quality of the LiPON samples. Notably, the composition of LiPON (the ratio of anions to P, Li content, and number of isolated O), lattice disorder, and defects are known to play a crucial role in Li^+^ diffusivity.^35^ Considering the induced disorder in the phosphate tetrahedra of LiPON due to the presence of N_a_ and N_d_ sites, it is possible that a similar phenomenon is at play in the melt-quench LiPON structures, which leads to its higher diffusivity compared to that of the equilibrated LiPON structures in our work. The presence of N_d_ sites could also contribute to this variation in diffusivity, as the equilibrated structures contain only N_a_ sites, compared to the melt-quench configurations that contain both N_a_ and N_d_ sites. This variation in how nitrogen is incorporated into different LiPON structures comes from the trained potential itself and the temperatures exposed during the MD runs. Nonetheless, the presence of N_d_ sites in the LiPON framework has been known to promote Li^+^ diffusivity^35^ attributed to Li^+^ being less tightly bound in the vicinity of N_d_ sites compared to N_a_ or O sites.

## Conclusions

Amorphous LiPON is an important class of Li solid electrolytes that are known to provide reasonable cycle life and power performance in thin-film devices while suppressing the growth of Li dendrites. In this work, we developed GNN-based NequIPs to investigate Li^+^ transport in bulk amorphous LiPON electrolytes and across a Li(110)||LiPON interface. We generated a DFT-based data set consisting of 13,454 structures and randomly split the data set into 90:10 training–validation sets to train and optimize the NequIPs. Note that our training data set comprised bulk and strained structures, Li-rich and Li-poor defective structures, LiPON-like amorphous configurations from AIMD, and slabs. Importantly, we observed the trained NequIPs, with equivariant tensor features, to be highly accurate with training (validation) energy and force MAEs of 5.5 (6.1) meV/atom and 13.6 (13.2) meV/ Å, respectively. Subsequently, we used the trained NequIPs to generate amorphous LiPON structures that exhibited N occupation of N_a_ and N_d_ sites, consistent with prior AIMD and experimental results. Our Li diffusivity estimates in bulk LiPON were qualitatively similar to those of previous studies as well, and we observed Li diffusivity to improve with increasing disorder in the LiPON structure. Further, we modeled Li^+^ transport across the Li(110)||LiPON interface, where we noticed Li movement across Li to LiPON (or vice versa) to be 1 order of magnitude slower than bulk motion within metallic Li and bulk LiPON. Thus, we do not expect the Li||LiPON interface to be insulating toward Li motion and any associated impedance buildup should be negligible. Finally, our study demonstrates that MLIPs can act as promising tools to model amorphous solid electrolytes as well as their associated interfacial behavior, and we hope our work instigates further studies exploring amorphous materials.

## Data Availability

All calculated data files and trained potentials are available to the public freely via our GitHub repository at https://github.com/sai-mat-group/ann-lipon.

## Supporting Information Available

Chemical and structural information about all the configurations used to create the data set; RDF plots for AIMD-generated melt-quench structures of Li_3_N, Li_3_P, and Li_2_O; details about the optimized hyperparameters used to train the NequIP potential; RDF plots for NequIP-generated LiPON structures at different temperatures; MSD vs Δ*t* plots for the Li(110)||LiPON interface at different temperatures (PDF)

## References

[ref1] Zhao Q.; Stalin S.; Zhao C.-Z.; Archer L. A. Designing Solid-State Electrolytes for Safe, Energy-Dense Batteries. Nat. Rev. Mater. 2020, 5 (3), 229–252. 10.1038/s41578-019-0165-5.

[ref2] Bachman J. C.; Muy S.; Grimaud A.; Chang H.-H.; Pour N.; Lux S. F.; Paschos O.; Maglia F.; Lupart S.; Lamp P.; Giordano L.; Shao-Horn Y. Inorganic Solid-State Electrolytes for Lithium Batteries: Mechanisms and Properties Governing Ion Conduction. Chem. Rev. 2016, 116 (1), 140–162. 10.1021/acs.chemrev.5b00563. 26713396

[ref3] Marbella L. E.; Zekoll S.; Kasemchainan J.; Emge S. P.; Bruce P. G.; Grey C. P. ^7^ Li NMR Chemical Shift Imaging To Detect Microstructural Growth of Lithium in All-Solid-State Batteries. Chem. Mater. 2019, 31 (8), 2762–2769. 10.1021/acs.chemmater.8b04875. 32051658 PMC7006347

[ref4] Su J.; Pasta M.; Ning Z.; Gao X.; Bruce P. G.; Grovenor C. R. M. Interfacial Modification between Argyrodite-Type Solid-State Electrolytes and Li Metal Anodes Using LiPON Interlayers. Energy Environ. Sci. 2022, 15 (9), 3805–3814. 10.1039/D2EE01390H.

[ref5] Jin Y.; Kneusels N.-J. H.; Magusin P. C. M. M.; Kim G.; Castillo-Martínez E.; Marbella L. E.; Kerber R. N.; Howe D. J.; Paul S.; Liu T.; Grey C. P. Identifying the Structural Basis for the Increased Stability of the Solid Electrolyte Interphase Formed on Silicon with the Additive Fluoroethylene Carbonate. J. Am. Chem. Soc. 2017, 139 (42), 14992–15004. 10.1021/jacs.7b06834. 28933161

[ref6] Szczuka C.; Karasulu B.; Groh M. F.; Sayed F. N.; Sherman T. J.; Bocarsly J. D.; Vema S.; Menkin S.; Emge S. P.; Morris A. J.; Grey C. P. Forced Disorder in the Solid Solution Li _3_ P–Li _2_ S: A New Class of Fully Reduced Solid Electrolytes for Lithium Metal Anodes. J. Am. Chem. Soc. 2022, 144 (36), 16350–16365. 10.1021/jacs.2c01913. 36040461 PMC9479069

[ref7] Li Y.; Song S.; Kim H.; Nomoto K.; Kim H.; Sun X.; Hori S.; Suzuki K.; Matsui N.; Hirayama M.; Mizoguchi T.; Saito T.; Kamiyama T.; Kanno R. A Lithium Superionic Conductor for Millimeter-Thick Battery Electrode. Science 2023, 381 (6653), 50–53. 10.1126/science.add7138. 37410839

[ref8] Song S.; Hori S.; Li Y.; Suzuki K.; Matsui N.; Hirayama M.; Saito T.; Kamiyama T.; Kanno R. Material Search for a Li _10_ GeP _2_ S _12_ -Type Solid Electrolyte in the Li–P–S–X (X = Br, I) System via Clarification of the Composition–Structure–Property Relationships. Chem. Mater. 2022, 34 (18), 8237–8247. 10.1021/acs.chemmater.2c01608.

[ref9] Hein A.; Martin J.; Schäfer M.; Weitzel K.-M. Electrodiffusion versus Chemical Diffusion in Alkali Calcium Phosphate Glasses: Implication of Structural Changes. J. Phys. Chem. C 2017, 121 (6), 3203–3211. 10.1021/acs.jpcc.6b11113.

[ref10] Ma C.; Chen K.; Liang C.; Nan C.-W.; Ishikawa R.; More K.; Chi M. Atomic-Scale Origin of the Large Grain-Boundary Resistance in Perovskite Li-Ion-Conducting Solid Electrolytes. Energy Environ. Sci. 2014, 7 (5), 1638 10.1039/c4ee00382a.

[ref11] Porz L.; Swamy T.; Sheldon B. W.; Rettenwander D.; Frömling T.; Thaman H. L.; Berendts S.; Uecker R.; Carter W. C.; Chiang Y. Mechanism of Lithium Metal Penetration through Inorganic Solid Electrolytes. Adv. Energy Mater. 2017, 7 (20), 1701003 10.1002/aenm.201701003.

[ref12] Raj V.; Venturi V.; Kankanallu V. R.; Kuiri B.; Viswanathan V.; Aetukuri N. P. B. Direct Correlation between Void Formation and Lithium Dendrite Growth in Solid-State Electrolytes with Interlayers. Nat. Mater. 2022, 21 (9), 1050–1056. 10.1038/s41563-022-01264-8. 35655030

[ref13] Nowak S.; Berkemeier F.; Schmitz G. Ultra-Thin LiPON Films – Fundamental Properties and Application in Solid State Thin Film Model Batteries. J. Power Sources 2015, 275, 144–150. 10.1016/j.jpowsour.2014.10.202.

[ref14] Bates J. B.; Dudney N. J.; Gruzalski G. R.; Zuhr R. A.; Choudhury A.; Luck C. F.; Robertson J. D. Electrical Properties of Amorphous Lithium Electrolyte Thin Films. Solid State Ion. 1992, 53–56, 647–654. 10.1016/0167-2738(92)90442-R.

[ref15] Herbert E. G.; Tenhaeff W. E.; Dudney N. J.; Pharr G. M. Mechanical Characterization of LiPON Films Using Nanoindentation. Thin Solid Films 2011, 520 (1), 413–418. 10.1016/j.tsf.2011.07.068.

[ref16] Han F.; Westover A. S.; Yue J.; Fan X.; Wang F.; Chi M.; Leonard D. N.; Dudney N. J.; Wang H.; Wang C. High Electronic Conductivity as the Origin of Lithium Dendrite Formation within Solid Electrolytes. Nat. Energy 2019, 4 (3), 187–196. 10.1038/s41560-018-0312-z.

[ref17] Bates J. B.; Lubben D.; Dudney N. J.; Hart F. X. 5 V Plateau in LiMn2 O 4 Thin Films. J. Electrochem. Soc. 1995, 142 (9), L149–L151. 10.1149/1.2048729.

[ref18] Bates J. B.; Dudney N. J.; Gruzalski G. R.; Zuhr R. A.; Choudhury A.; Luck C. F.; Robertson J. D. Fabrication and Characterization of Amorphous Lithium Electrolyte Thin Films and Rechargeable Thin-Film Batteries. J. Power Sources 1993, 43 (1–3), 103–110. 10.1016/0378-7753(93)80106-Y.

[ref19] Fleutot B.; Pecquenard B.; Martinez H.; Letellier M.; Levasseur A. Investigation of the Local Structure of LiPON Thin Films to Better Understand the Role of Nitrogen on Their Performance. Solid State Ion. 2011, 186 (1), 29–36. 10.1016/j.ssi.2011.01.006.

[ref20] Roh N.-S.; Lee S.-D.; Kwon H.-S. Effects of Deposition Condition on the Ionic Conductivity and Structure of Amorphous Lithium Phosphorus Oxynitrate Thin Film. Scr. Mater. 1999, 42 (1), 43–49. 10.1016/S1359-6462(99)00307-3.

[ref21] Day D. E. Structural Role of Nitrogen in Phosphate Glasses. J. Non-Cryst. Solids 1989, 112 (1–3), 7–14. 10.1016/0022-3093(89)90488-2.

[ref22] Jacke S.; Song J.; Dimesso L.; Brötz J.; Becker D.; Jaegermann W. Temperature Dependent Phosphorous Oxynitride Growth for All-Solid-State Batteries. J. Power Sources 2011, 196 (16), 6911–6914. 10.1016/j.jpowsour.2010.12.022.

[ref23] Marchand R. Nitrogen-Containing Phosphate Glasses. J. Non-Cryst. Solids 1983, 56 (1–3), 173–178. 10.1016/0022-3093(83)90464-7.

[ref24] Wang B.; Kwak B. S.; Sales B. C.; Bates J. B. Ionic Conductivities and Structure of Lithium Phosphorus Oxynitride Glasses. J. Non-Cryst. Solids 1995, 183 (3), 297–306. 10.1016/0022-3093(94)00665-2.

[ref25] Wang B.; Bates J. B.; Hart F. X.; Sales B. C.; Zuhr R. A.; Robertson J. D. Characterization of Thin-Film Rechargeable Lithium Batteries with Lithium Cobalt Oxide Cathodes. J. Electrochem. Soc. 1996, 143 (10), 3203–3213. 10.1149/1.1837188.

[ref26] Li J.; Lai W. Structure and Ionic Conduction Study on Li3PO4 and LiPON (Lithium Phosphorous Oxynitride) with the Density-Functional Tight-Binding (DFTB) Method. Solid State Ion. 2020, 351, 115329 10.1016/j.ssi.2020.115329.

[ref27] Yu X.; Bates J. B.; Jellison G. E.; Hart F. X. A Stable Thin-Film Lithium Electrolyte: Lithium Phosphorus Oxynitride. J. Electrochem. Soc. 1997, 144 (2), 524–532. 10.1149/1.1837443.

[ref28] Li J.; Ma C.; Chi M.; Liang C.; Dudney N. J. Solid Electrolyte: The Key for High-Voltage Lithium Batteries. Adv. Energy Mater. 2015, 5 (4), 1401408 10.1002/aenm.201401408.

[ref29] Magistris A.; Chiodelli G.; Duclot M. Silver Borophosphate Glasses: Ion Transport, Thermal Stability and Electrochemical Behaviour. Solid State Ion. 1983, 9–10, 611–615. 10.1016/0167-2738(83)90303-X.

[ref30] Deng Y.; Eames C.; Chotard J.-N.; Lalère F.; Seznec V.; Emge S.; Pecher O.; Grey C. P.; Masquelier C.; Islam M. S. Structural and Mechanistic Insights into Fast Lithium-Ion Conduction in Li _4_ SiO _4_ – Li _3_ PO _4_ Solid Electrolytes. J. Am. Chem. Soc. 2015, 137 (28), 9136–9145. 10.1021/jacs.5b04444. 26118319

[ref31] Wang B.; Chakoumakos B. C.; Sales B. C.; Kwak B. S.; Bates J. B. Synthesis, Crystal Structure, and Ionic Conductivity of a Polycrystalline Lithium Phosphorus Oxynitride with the γ-Li3PO4 Structure. J. Solid State Chem. 1995, 115 (2), 313–323. 10.1006/jssc.1995.1140.

[ref32] Pickard C. J.; Mauri F. All-Electron Magnetic Response with Pseudopotentials: NMR Chemical Shifts. Phys. Rev. B 2001, 63 (24), 245101 10.1103/PhysRevB.63.245101.

[ref33] Nimisha C. S.; Rao K. Y.; Venkatesh G.; Rao G. M.; Munichandraiah N. Sputter Deposited LiPON Thin Films from Powder Target as Electrolyte for Thin Film Battery Applications. Thin Solid Films 2011, 519 (10), 3401–3406. 10.1016/j.tsf.2011.01.087.

[ref34] Sicolo S.; Albe K. First-Principles Calculations on Structure and Properties of Amorphous Li5P4O8N3 (LiPON). J. Power Sources 2016, 331, 382–390. 10.1016/j.jpowsour.2016.09.059.

[ref35] Lacivita V.; Artrith N.; Ceder G. Structural and Compositional Factors That Control the Li-Ion Conductivity in LiPON Electrolytes. Chem. Mater. 2018, 30 (20), 7077–7090. 10.1021/acs.chemmater.8b02812.

[ref36] Lacivita V.; Westover A. S.; Kercher A.; Phillip N. D.; Yang G.; Veith G.; Ceder G.; Dudney N. J. Resolving the Amorphous Structure of Lithium Phosphorus Oxynitride (Lipon). J. Am. Chem. Soc. 2018, 140 (35), 11029–11038. 10.1021/jacs.8b05192. 30036061

[ref37] Marple M. A. T.; Wynn T. A.; Cheng D.; Shimizu R.; Mason H. E.; Meng Y. S. Local Structure of Glassy Lithium Phosphorus Oxynitride Thin Films: A Combined Experimental and Ab Initio Approach. Angew. Chem., Int. Ed. 2020, 59 (49), 22185–22193. 10.1002/anie.202009501. 32818306

[ref38] Choyal V.; Sagar N.; Sai Gautam G. Constructing and Evaluating Machine-Learned Interatomic Potentials for Li-Based Disordered Rocksalts. J. Chem. Theory Comput. 2024, 20 (11), 4844–4856. 10.1021/acs.jctc.4c00039. 38787289

[ref39] Maxson T.; Szilvási T. Transferable Water Potentials Using Equivariant Neural Networks. J. Phys. Chem. Lett. 2024, 15 (14), 3740–3747. 10.1021/acs.jpclett.4c00605. 38547514

[ref40] Ghaffari K.; Bavdekar S.; Spearot D. E.; Subhash G. Validation Workflow for Machine Learning Interatomic Potentials for Complex Ceramics. Comput. Mater. Sci. 2024, 239, 112983 10.1016/j.commatsci.2024.112983.

[ref41] Sosso G. C.; Miceli G.; Caravati S.; Behler J.; Bernasconi M. Neural Network Interatomic Potential for the Phase Change Material GeTe. Phys. Rev. B 2012, 85 (17), 174103 10.1103/PhysRevB.85.174103.

[ref42] Deringer V. L.; Caro M. A.; Csányi G. Machine Learning Interatomic Potentials as Emerging Tools for Materials Science. Adv. Mater. 2019, 31 (46), 1902765 10.1002/adma.201902765. 31486179

[ref43] Fu X.; Wu Z.; Wang W.; Xie T.; Keten S.; Gomez-Bombarelli R.; Jaakkola T. Forces Are Not Enough: Benchmark and Critical Evaluation for Machine Learning Force Fields with Molecular Simulations. arXiv 2022, 10.48550/arXiv.2210.07237.

[ref44] Schütt K. T.; Sauceda H. E.; Kindermans P.-J.; Tkatchenko A.; Müller K.-R. SchNet – A Deep Learning Architecture for Molecules and Materials. J. Chem. Phys. 2018, 148 (24), 241722 10.1063/1.5019779. 29960322

[ref45] Xie T.; Grossman J. C. Crystal Graph Convolutional Neural Networks for an Accurate and Interpretable Prediction of Material Properties. Phys. Rev. Lett. 2018, 120 (14), 145301 10.1103/PhysRevLett.120.145301. 29694125

[ref46] Batzner S.; Musaelian A.; Sun L.; Geiger M.; Mailoa J. P.; Kornbluth M.; Molinari N.; Smidt T. E.; Kozinsky B. E(3)-Equivariant Graph Neural Networks for Data-Efficient and Accurate Interatomic Potentials. Nat. Commun. 2022, 13 (1), 2453 10.1038/s41467-022-29939-5. 35508450 PMC9068614

[ref47] Ong S. P.; Richards W. D.; Jain A.; Hautier G.; Kocher M.; Cholia S.; Gunter D.; Chevrier V. L.; Persson K. A.; Ceder G. Python Materials Genomics (Pymatgen): A Robust, Open-Source Python Library for Materials Analysis. Comput. Mater. Sci. 2013, 68, 314–319. 10.1016/j.commatsci.2012.10.028.

[ref48] Geiger M.; Smidt T. E3nn: Euclidean Neural Networks. arXiv 2022, 10.48550/arXiv.2210.07237.

[ref49] Thomas N.; Smidt T.; Kearnes S.; Yang L.; Li L.; Kohlhoff K.; Riley P. Tensor Field Networks: Rotation- and Translation-Equivariant Neural Networks for 3D Point Clouds. arXiv 2018, 10.48550/arXiv.1802.08219.

[ref50] Kresse G.; Furthmüller J. Efficiency of Ab-Initio Total Energy Calculations for Metals and Semiconductors Using a Plane-Wave Basis Set. Comput. Mater. Sci. 1996, 6 (1), 15–50. 10.1016/0927-0256(96)00008-0.

[ref51] Kresse G.; Furthmüller J. Efficient Iterative Schemes for *Ab Initio* Total-Energy Calculations Using a Plane-Wave Basis Set. Phys. Rev. B 1996, 54 (16), 11169–11186. 10.1103/PhysRevB.54.11169. 9984901

[ref52] Perdew J. P.; Burke K.; Ernzerhof M. Generalized Gradient Approximation Made Simple. Phys. Rev. Lett. 1996, 77 (18), 3865–3868. 10.1103/PhysRevLett.77.3865. 10062328

[ref53] Kresse G.; Joubert D. From Ultrasoft Pseudopotentials to the Projector Augmented-Wave Method. Phys. Rev. B 1999, 59 (3), 1758–1775. 10.1103/PhysRevB.59.1758.

[ref54] Hoover W. G. Canonical Dynamics: Equilibrium Phase-Space Distributions. Phys. Rev. A 1985, 31 (3), 1695–1697. 10.1103/PhysRevA.31.1695. 9895674

[ref55] Nosé S. A Unified Formulation of the Constant Temperature Molecular Dynamics Methods. J. Chem. Phys. 1984, 81 (1), 511–519. 10.1063/1.447334.

[ref56] Nosé S. Constant Temperature Molecular Dynamics Methods. Prog. Theor. Phys. Suppl. 1991, 103, 1–46. 10.1143/PTPS.103.1.

[ref57] Thompson A. P.; Aktulga H. M.; Berger R.; Bolintineanu D. S.; Brown W. M.; Crozier P. S.; in't Veld P. J.; Kohlmeyer A.; Moore S. G.; Nguyen T. D.; Shan R.; Stevens M. J.; Tranchida J.; Trott C.; Plimpton S. J. LAMMPS - a Flexible Simulation Tool for Particle-Based Materials Modeling at the Atomic, Meso, and Continuum Scales. Comput. Phys. Commun. 2022, 271, 108171 10.1016/j.cpc.2021.108171.

[ref58] He X.; Zhu Y.; Epstein A.; Mo Y. Statistical Variances of Diffusional Properties from Ab Initio Molecular Dynamics Simulations. Npj Comput. Mater. 2018, 4 (1), 18 10.1038/s41524-018-0074-y.

[ref59] Ivanov-Shitz A. K.; Kireev V. V.; Mel’nikov O. K.; Demianets L. N. Growth and Ionic Conductivity of γ-Li3PO4. Crystallogr. Rep. 2001, 46 (5), 864–867. 10.1134/1.1405880.

[ref60] Kuwata N.; Lu X.; Miyazaki T.; Iwai Y.; Tanabe T.; Kawamura J. Lithium Diffusion Coefficient in Amorphous Lithium Phosphate Thin Films Measured by Secondary Ion Mass Spectroscopy with Isotope Exchange Methods. Solid State Ion. 2016, 294, 59–66. 10.1016/j.ssi.2016.06.015.

[ref61] Wang J.; Panchal A. A.; Sai Gautam G.; Canepa P. The Resistive Nature of Decomposing Interfaces of Solid Electrolytes with Alkali Metal Electrodes. J. Mater. Chem. A 2022, 10 (37), 19732–19742. 10.1039/D2TA02202H.

[ref62] Su Y.; Falgenhauer J.; Polity A.; Leichtweiß T.; Kronenberger A.; Obel J.; Zhou S.; Schlettwein D.; Janek J.; Meyer B. K. LiPON Thin Films with High Nitrogen Content for Application in Lithium Batteries and Electrochromic Devices Prepared by RF Magnetron Sputtering. Solid State Ion. 2015, 282, 63–69. 10.1016/j.ssi.2015.09.022.

[ref63] Deng B.; Zhong P.; Jun K.; Riebesell J.; Han K.; Bartel C. J.; Ceder G. CHGNet as a Pretrained Universal Neural Network Potential for Charge-Informed Atomistic Modelling. Nat. Mach. Intell. 2023, 5 (9), 1031–1041. 10.1038/s42256-023-00716-3.

[ref64] Musaelian A.; Batzner S.; Johansson A.; Sun L.; Owen C. J.; Kornbluth M.; Kozinsky B. Learning Local Equivariant Representations for Large-Scale Atomistic Dynamics. Nat. Commun. 2023, 14 (1), 579 10.1038/s41467-023-36329-y. 36737620 PMC9898554

[ref65] Behler J.; Parrinello M. Generalized Neural-Network Representation of High-Dimensional Potential-Energy Surfaces. Phys. Rev. Lett. 2007, 98 (14), 146401 10.1103/PhysRevLett.98.146401. 17501293

[ref66] Batatia I.; Kovács D. P.; Simm G. N. C.; Ortner C.; Csányi G. MACE: Higher Order Equivariant Message Passing Neural Networks for Fast and Accurate Force Fields. arXiv 2023, 10.48550/arXiv.2206.07697.

[ref67] Wang K.; Janek J.; Mollenhauer D. Insight into the Li/LiPON Interface at the Molecular Level: Interfacial Decomposition and Reconfiguration. Chem. Mater. 2024, 36, 5133 10.1021/acs.chemmater.4c00377.

