# Supplementary material for: Investigating Ionic Diffusivity in Amorphous LiPON using Machine-Learned Interatomic Potentials
Source: ACS Mater Au. 2025 Feb 5;5(3):458–68. doi: 10.1021/acsmaterialsau.4c00117 (PMC12082356; doi:10.1021/acsmaterialsau.4c00117)
Supplement: Supplementary file 1 — mg4c00117_si_001.pdf [file mg4c00117_si_001.pdf]

Supplementary Information

Investigating Ionic Diffusivity in Amorphous LiPON using Machine Learned Interatomic Potentials

Aqshat Seth, Rutvij Pankaj Kulkarni, Gopalakrishnan Sai Gautam*

Department of Materials Engineering, Indian Institute of Science, Bengaluru 560012, India

*Corresponding author: saigautamg@iisc.ac.in

Dataset Generation

The unit cells for each of the 19 systems shown in Table 1. were relaxed and used to generate the rest of the dataset using the pymatgen[1] package. Further information on each category of structures and the number of structures in each category can be found below:

Table S1. Initial set of structures used to generate the DFT-based dataset.

Chemical Systems
Li
Li_2O
Li_2O_2
LiO_8
LiP
Li_3P
LiP_7
Li_3P_7
Li_3N
LiN_3
$LiPN_2$
Li_3PO_4
$Li_4P_2O_7$
Li_7PN_4
Li_2PNO_2
P_3N_5
P_2O_5
PNO
$LiPN_2$

Hydrostatic Strain: 306 structures were generated by uniformly applying strains along the lattice directions with an increment of 1% volumetric change between the initial bulk structures and the hydrostatically strained structures starting from -10% and going upto +9%.

Orthorhombic Strain: 328 structures were generated by uniformly applying strains along one lattice direction, while keeping the other two lattice parameters fixed with an increment of 1% volumetric change between the initial bulk structures and the orthorhombically strained structures starting from -10% and going upto +9%.

Monoclinic Strain: 252 structures were generated by uniformly changing one of the angles of the initial bulk structures while keeping the volume of the structure unchanged, with an incremental change of 10%.

Slabs: Slabs were constructed with a vacuum spacing of 20 Å and slab thicknesses between 10 Å to 30 Å. 72 slabs from elemental Li, 530 slabs from Li_3P, 252 slabs from Li_2O_2, 179 slabs from Li_2O and 186 slabs from Li_3N were added to the dataset.

Defective Structures: The AIMD-based Li-rich and Li-poor supercell structures were generated at a composition of $Li_{3.04}PN_{0.046}O_{3.95}$ (39 structures) and $Li_{2.95}PN_{0.046}O_{3.91}$ (88 structures) respectively. The Li-rich and Li-poor unit cell structures were generated at compositions of $Li_{3.5}PN_{0.5}O_{3.5}$ (40 structures) and $Li_{2.5}PN_{0.5}O_3$ (89 structures) respectively. The structures generated by enumerating the removal of 1 P and the concomitant addition of 5 Li within Li_7PN_4 2x2x2 supercells were at a composition of $Li_{8.71}PN_{4.57}$ (74 structures)

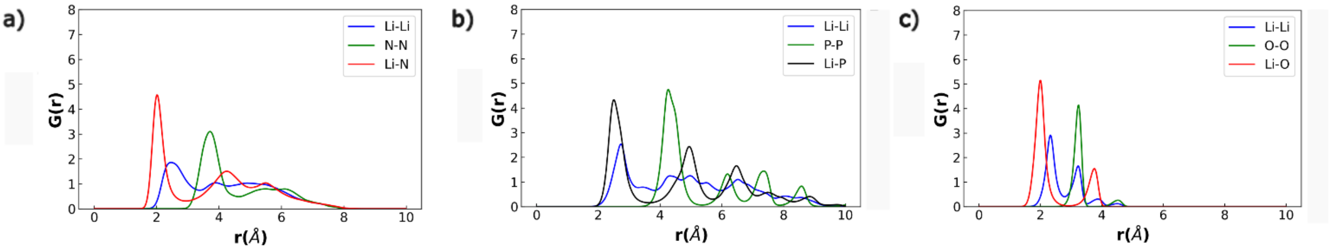

Figure S1. Radial distribution function (RDF) of ab initio molecular dynamics (AIMD) generated melt-quench structures of a) Li_3N, b) Li_3P, and c) Li_2O. The RDFs displayed are calculated in the quenched structure at 250 K, obtained after being heated to 1000 K in the case of Li_3N and 2000 K for Li_3P and Li_2O.

Table S2. Hyperparameter values used for training the neural equivariant interatomic potential (NequIP) model. The training energy and force MAE were found to be of 5.5 meV/atom and 13.6 meV/Å, respectively with the validation energy and force MAE being 6.1 meV/atom and 13.2 meV/Å respectively.

**Hyperparameter**	**Optimized Value**
Cutoff radius	4.3 Å
Interaction blocks	6
Tensor product layers per interaction block	2
Neurons in each product layer	64
Basis function in trainable basis set	8
Polynomial cutoff for envelope function	6 Å
Learning rate	0.0044

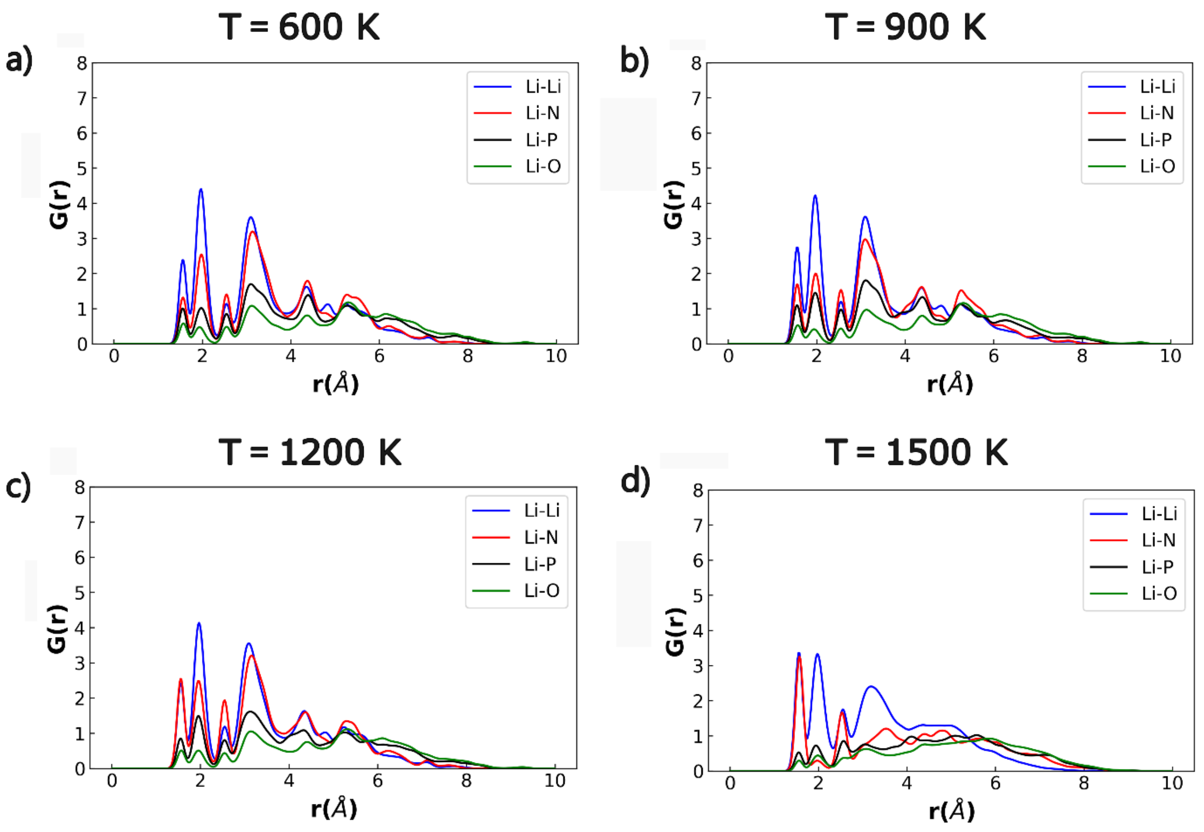

Figure S2. Time-averaged RDFs upon quenching to 250 K for NequIP-generated amorphous LiPON structures. The structure is initially melted and quenched to a) 600 K, b) 900 K, c) 1200 K and d) 1500 K, before being finally quenched to 250 K.

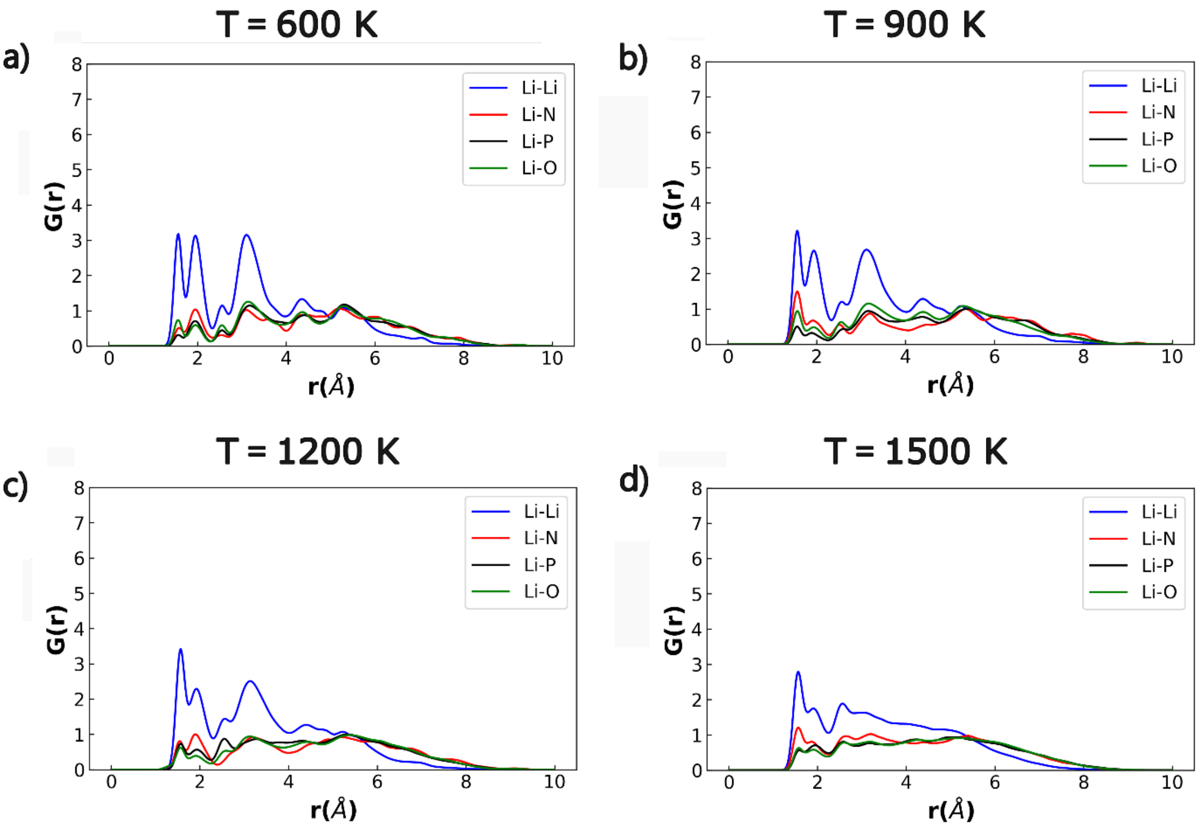

Figure S3. Time-averaged RDFs at 250 K for NequIP-based amorphous LiPON structures. The structures are initially equilibrated at a) 600 K, b) 900 K, c) 1200 K and d) 1500 K before being quenched to 250 K.

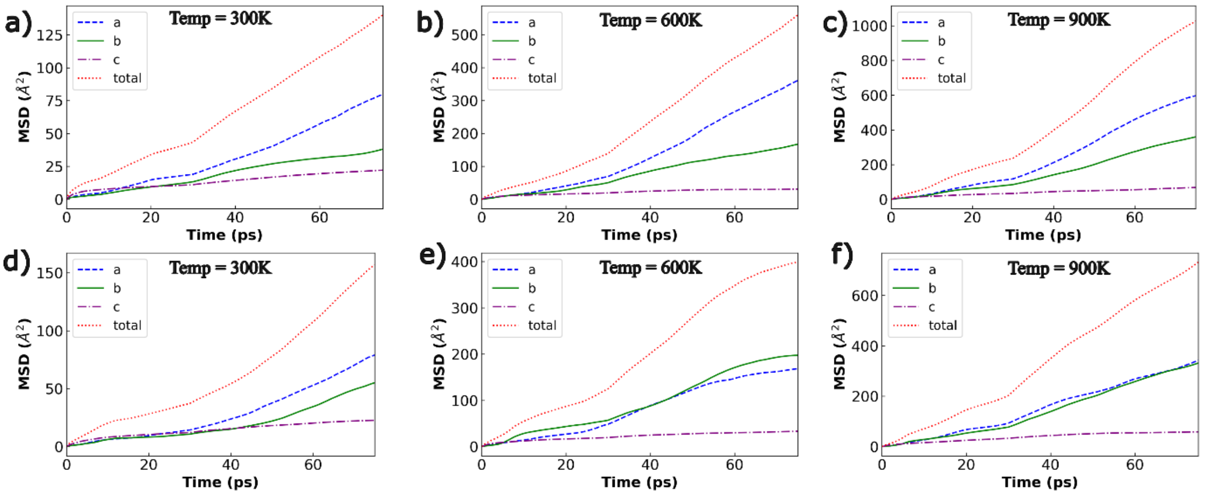

Figure S4. Mean square displacement (MSD) versus time-step (Δt) plots for the Li(110)||LiPON and Li(111)||LiPON interfaces in the directions of the lattice parameters and the total MSD. Plots represent calculations at 300 K, 600 K and 900 K, with a), b) and c) representing the Li(110)||LiPON interface and d), e) and f) representing the Li(111)||LiPON interface . Here, *c* refers to the perpendicular direction across the interface.

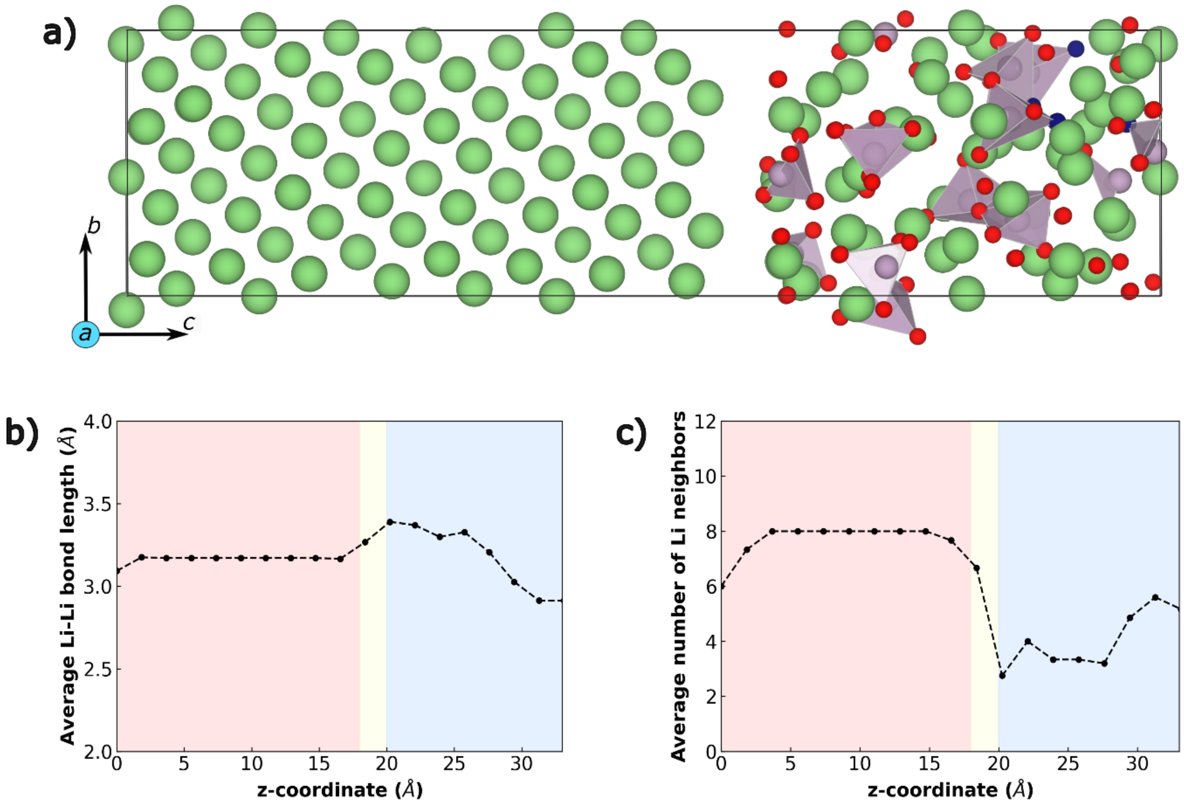

Figure S5. a) The Li-(111)||LiPON interface. b) Variation in the average Li-Li bond length and c) variation in the average number of Li neighbors along the *c*-axis.

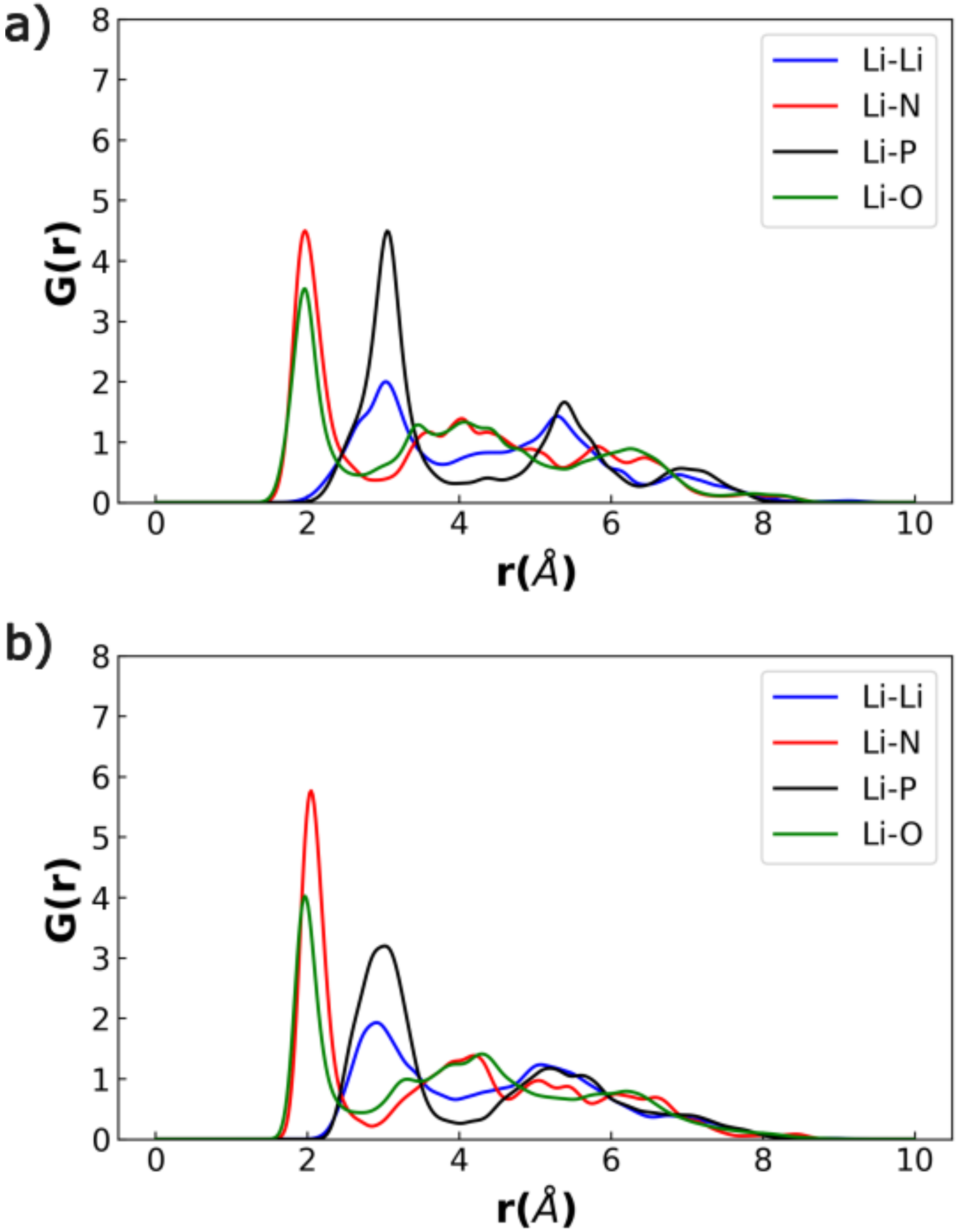

Figure S6. Comparisons of the time-averaged RDFs of a) AIMD generated LiPON and b) Melt-quench based NequIP generated LiPON.

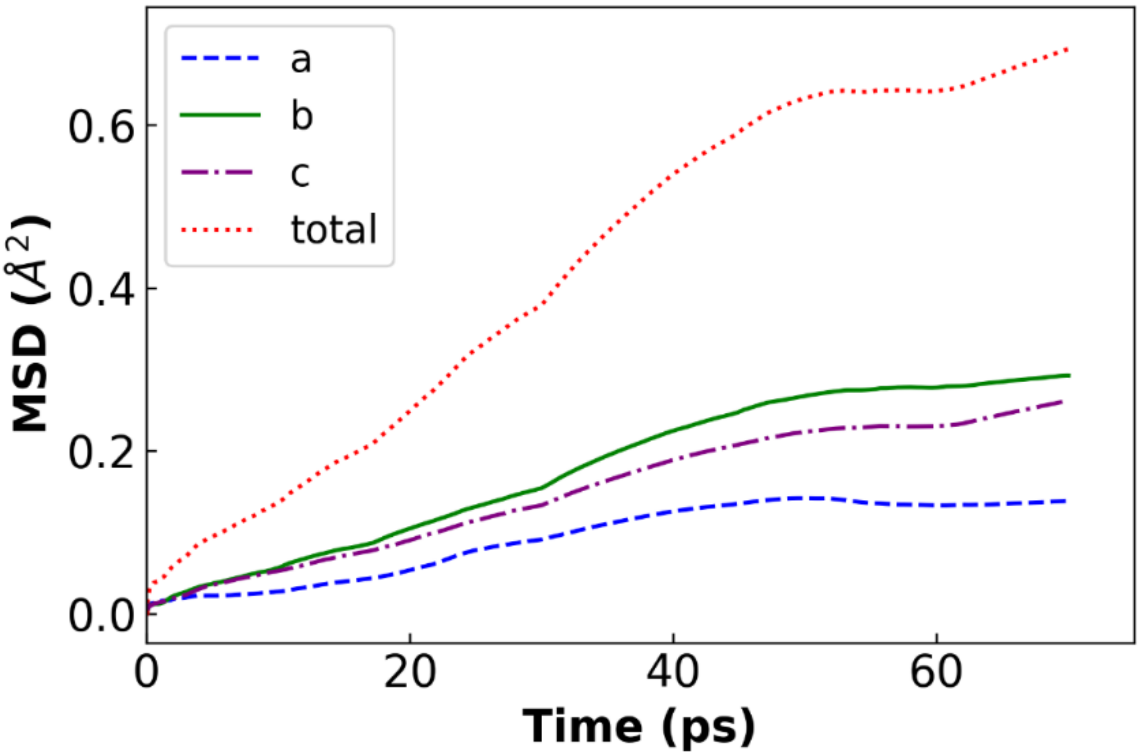

Figure S7. Mean square displacement (MSD) versus time-step (Δt) plots for the melt-quench configuration generated via NequIP at 300 K.

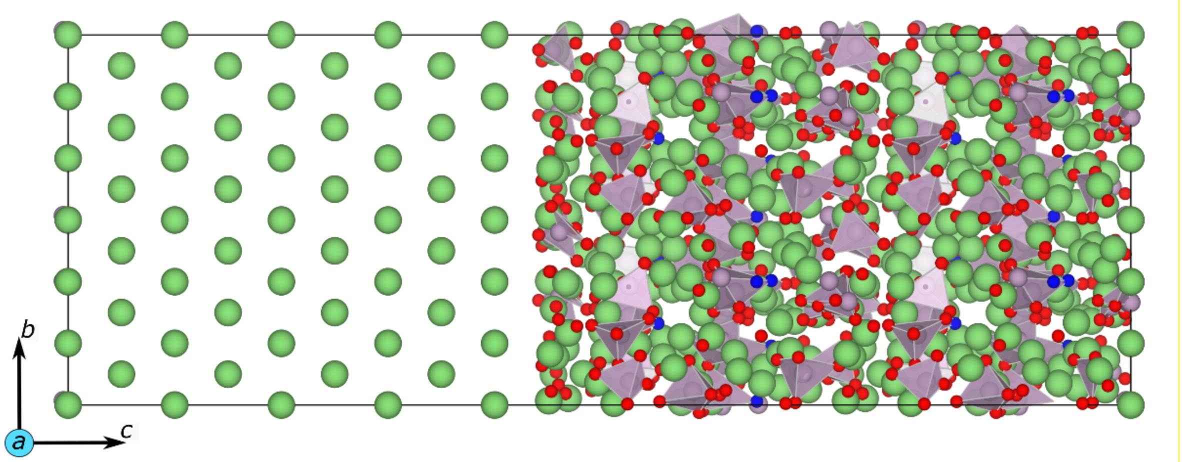

Figure S8. Li(110)||LiPON interface consisting of 1316 atoms.

References

(1) Ong, S. P.; Richards, W. D.; Jain, A.; Hautier, G.; Kocher, M.; Cholia, S.; Gunter, D.; Chevrier, V. L.; Persson, K. A.; Ceder, G. Python Materials Genomics (Pymatgen): A Robust, Open-Source Python Library for Materials Analysis. *Comput. Mater. Sci.* **2013**, *68*, 314–319. https://doi.org/10.1016/j.commatsci.2012.10.028.
